# A Lysosome‐Targeted Magnetic Nanotorquer Mechanically Triggers Ferroptosis for Breast Cancer Treatment

**DOI:** 10.1002/advs.202302093

**Published:** 2023-12-14

**Authors:** Xueyan Wei, Yingze Li, Haotian Chen, Rui Gao, Peng Ning, Yingying Wang, Wanxin Huang, Erzhen Chen, Lan Fang, Xingrong Guo, Cheng Lv, Yu Cheng

**Affiliations:** ^1^ Translational Research Institute of Brain and Brain‐Like Intelligence Shanghai Fourth People's Hospital, School of Medicine Tongji University Shanghai 200092 China; ^2^ Shanghai Tenth People's Hospital, School of Medicine Tongji University Cancer Center Shanghai 200072 China; ^3^ Hubei Key Laboratory of Embryonic Stem Cell Research, Hubei Clinical Research Center for Umbilical Cord Blood Hematopoietic Stem Cells Taihe Hospital Hubei University of Medicine Shiyan Hubei 442000 China

**Keywords:** cancer treatment, ferroptosis inducer, labile iron pool, magnetic nanotorquer, mechanical regulation

## Abstract

Targeting ferroptosis has attracted exponential attention to eradicate cancer cells with high iron‐dependent growth. Increasing the level of intracellular labile iron pool via small molecules and iron‐containing nanomaterials is an effective approach to induce ferroptosis but often faces insufficient efficacy due to the fast drug metabolism and toxicity issues on normal tissues. Therefore, developing a long‐acting and selective approach to regulate ferroptosis is highly demanded in cancer treatment. Herein, a lysosome‐targeted magnetic nanotorquer (T7‐MNT) is proposed as the mechanical tool to dynamically induce the endogenous Fe^2+^ pool outbreak for ferroptosis of breast cancer. T7‐MNTs target lysosomes via the transferrin receptor‐mediated endocytosis in breast cancer cells. Under the programmed rotating magnetic field, T7‐MNTs generate torques to trigger endogenous Fe^2+^ release by disrupting the lysosomal membrane. This magneto‐mechanical manipulation can induce oxidative damage and antioxidant defense imbalance to boost frequency‐ and time‐dependent lipid peroxidization. Importantly, in vivo studies show that T7‐MNTs can efficiently trigger ferroptosis under the magnetic field and play as a long‐acting physical inducer to boost ferrotherapy efficacy in combination with RSL3. It is anticipated that this dynamic targeted strategy can be coupled with current ferroptosis inducers to achieve enhanced efficacy and inspire the design of mechanical‐based ferroptosis inducers for cancer treatment.

## Graphical abstract

Under the programmed magnetic field stimulation, the lysosome‐targeted magnetic nanotorquer (T7‐MNTs) generated torques to disrupt the lysosomal membrane and dynamically induce the endogenous Fe^2+^ pool outbreak. T7‐MNTs induced oxidative damage and antioxidant defense imbalance to trigger frequency‐ and time‐dependent ferroptosis, and played as an effective physical inducer for anticancer treatment.

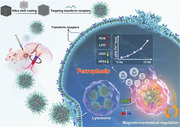

## 1 Introduction

As an iron‐dependent cell death process, ferroptosis is distinct from apoptosis, necrosis, and autophagy due to its unique morphological, genetic, and biochemical characteristics, which has been regarded as an innovative therapeutic strategy for cancer treatment.^[^ ^1^ ^]^ Labile ferrous ions (Fe^2+^) pool plays a fundamental role in ferroptosis.^[^ ^2^ ^]^ As an intracellular redox‐active metal, the labile Fe^2+^ could directly catalyze lipid peroxidation via Fenton reaction and serve as a key factor to affect the activity of enzymes such as lipoxygenases (LOXs) and nicotinamide adenine dinucleotide phosphate hydride (NADPH) oxidases (NOXs), which can fuel toxic lipid peroxides production.^[^ ^3^ ^]^ Among different cellular compartments, lysosomes are the emerging ferroptosis targets due to the capability to tune the iron metabolism.^[^ ^4^ ^]^ Instead of the established role as a digestive compartment of cells, lysosomes store a pool of redox‐active iron and are unusually sensitive to the oxidative stress. Small molecules have been developed to trigger the iron release by degradation of ferritin in lysosomes and induce the sequential reactive oxygen species (ROS) production by lysosomal membrane permeabilization.^[^ ^5^ ^]^ However, their therapeutic effect is often compromised in vivo by fast drug metabolism, insufficient ROS generation and unwanted toxicity in normal tissues.

Nanomaterials can be enriched in lysosomes by the endocytosis mechanism of cancer cells, which offers an alternative strategy for targeting ferroptosis. Taking the advantage of acidic environment in lysosomes, iron‐containing nanomaterials with the pH‐responsive property have been designed to release the exogenous iron and generate ROS to activate ferroptosis in cancer cells.^[^ ^6^ ^]^ To enhance ferroptosis efficacy and prevent the unwanted side effects in treating cancers, functional nanomaterials with versatile compositions coupled with physical triggers become promising platforms to accelerate ROS generation and induce the on‐demand ferroptosis in a spatiotemporal and selective fashion.^[^ ^7^ ^]^ Recently, photothermal‐responsive nanomaterials have shown the capability to cause heat‐induced lysosome dysfunction and generate ROS to damage cancer cells via ferroptosis.^[^ ^8^ ^]^ Ultrasound stimulation is also proved to be an effective approach to control the sonosensitizer‐embedded nanocluster which could generate highly reactive ^1^O_2_ and promote the accumulation of lipid peroxide species to initiate ferroptosis.^[^ ^9^ ^]^ Although these advances are encouraging, how to precisely trigger the endogenous labile iron release from lysosomes with no tissue penetration limit remains a great challenge in targeted ferroptosis of cancer therapy.

The magneto‐mechanical approach, coupled by magnetic nanomaterials and alternating magnetic fields with low frequency, is an emerging physical modality for cancer treatment.^[^ ^10^ ^]^ Under the magnetic field, magnetic nanomaterials could transform the energy in the form of torques or forces to remotely and quantitatively regulate cell biological functions. Deep and precise control at the nanoscale can be achieved via multiple mechanisms including receptor‐ligand interaction,^[^ ^11^ ^]^ ion channel activation,^[^ ^12^ ^]^ cytoskeleton disruption,^[^ ^13^ ^]^ and cell membrane damage.^[^ ^14^ ^]^ It is shown that the low frequency magnetic field treatment could deform hybrid vesicles containing iron oxide nanoparticles and trigger drug release for cancer cell death.^[^ ^15^ ^]^ Several reports also proved that iron oxide nanoparticles can cause lysosome dysfunction and trigger apoptosis of cancer cells under dynamic magnetic fields.^[^ ^16^ ^]^ While this strategy has attached the most attention to elucidate the conventional cell death pathways, whether ferroptosis could be directly triggered via this mechanical approach remains unexplored. Developing lysosomal‐targeted magneto‐mechanical ferroptosis inducers will be essential for achieving the long‐acting and selective cancer cell destruction for deep‐seated tumors.

**Scheme 1:**
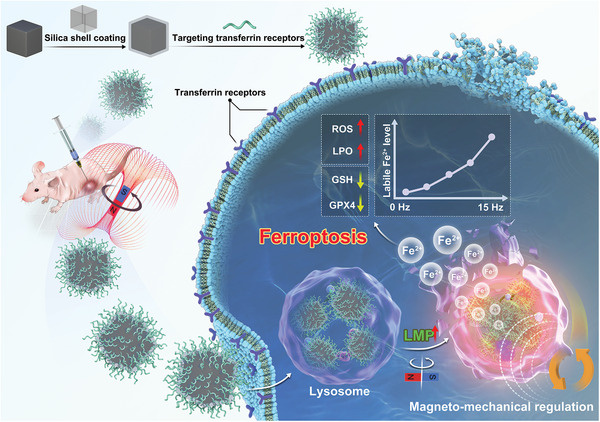
Illustration of lysosome‐targeted magnetic nanotorquers dynamically triggered the endogenous Fe^2+^ pool outbreak for evoking the ferroptosis in a programmable manner by locally disrupting the lysosomal membrane under MF.

Herein, we proposed a lysosome‐targeted magnetic nanotorquer (MNT) as the mechanical tool to dynamically induce the endogenous Fe^2+^ pool outbreak for ferroptosis of breast cancer. The 60 nm cubic shape magnetic nanoparticles (Zn_0.4_Fe_2.6_O_4_) were coated with silicon oxide (SiO_2_) shell to prevent the exogenous iron from leaching in acidic biological environments and realize the direct mechanical regulation. To achieve the cancer selectivity and subcellular localization, the T7 peptide, which targets the transferrin receptor on breast cancer cells, was modified on the silica shell to obtain the lysosome‐targeted MNT (T7‐MNT). As shown in **Scheme** **1**, under a rotating magnetic field (MF), the T7‐MNTs inside lysosomes of breast cancer cells could generate the torques, which could spatially impair the lysosomal membrane structure and trigger the endogenous labile Fe^2+^ release from lysosomes into the cytosol in a programmable way. The released Fe^2+^ could further enhance ROS generation and catalyze intracellular lipid peroxidation, leading to ferroptosis. The in vivo ferrotherapy effect of T7‐MNTs was evaluated in mice bearing breast cancer. We believe that the T7‐MNTs will promote the development of precise ferroptosis control and will further drive the clinical developments of ferroptosis‐based cancer therapy.

## 2 Results and Discussion

### 2.1 Synthesis and Characterization of T7‐MNTs

**Figure 1:**
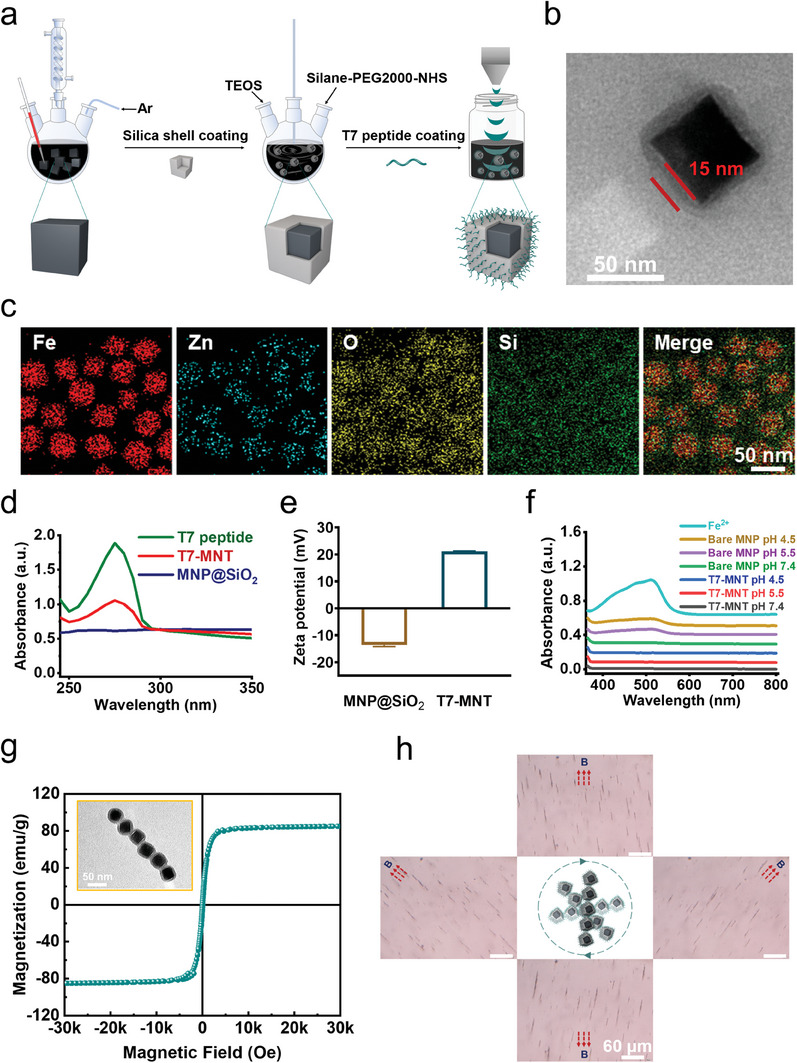
Design and characterization of T7‐MNTs. a) Scheme illustration of T7‐MNTs synthetic procedures. (b) TEM images of T7‐MNTs (scale bar: 50 nm, 15 nm). c) Elemental mapping analysis and element distribution of T7‐MNTs (scale bar: 50 nm). d) Ultraviolet absorption spectra of T7 peptide, T7‐MNTs, and MNP@SiO_2_. e) Zeta potential of MNP@SiO_2_ and T7‐MNTs. f) Iron release of bare MNPs and T7‐MNTs in different pH conditions. g) Magnetic properties of T7‐MNTs represented by M‐H curves. h) Optical images of T7‐MNTs swarms rotated with the magnetic field at 40 mT and 0.5 Hz (scale bar: 60 µm).

The cubic iron oxide nanoparticle was chosen as the intracellular MNT template. As shown in **Figure** **1**a, the 60 nm zinc‐doped magnetic nanoparticle (MNP, Zn_0.4_Fe_2.6_O_4_) in the cubic shape was synthesized using the thermal decomposition method.^[^ ^17^ ^]^ To prevent exogenous iron leaking and obtain stability in the aqueous solution, the MNPs were sequentially reacted with tetraethyl orthosilicate (TEOS) and silane‐PEG2000‐NHS to form a SiO_2_ shell with a 15 nm coating thickness (MNP@SiO_2_) (Figure 1b). The composition of MNP@SiO_2_ was determined by elemental mapping analysis, showing that each element was distributed in the framework of MNP@SiO_2_ with a high uniformity (Figure 1c). To endow the nanoparticles with targeting ability to cancer cells, T7 peptide (7 peptide, HAIYPRH, histidine‐alanine‐isoleucine‐tyrosine‐proline‐arginine‐histidine), which could target to transferrin receptors selectively,^[^ ^18^ ^]^ was conjugated to the SiO_2_ shell surface (T7‐MNT). As displayed in Figure 1d, T7‐MNTs showed a strong absorption peak of T7 peptide at ≈275 nm, while no such peak was found in the MNP@SiO_2_. Furthermore, the average zeta potential of T7‐MNTs was 21.2 ± 0.1 mV, significantly higher than that of the MNP@SiO_2_ (−13.6 ± 0.6 mV) (Figure 1e). The average hydrodynamic diameter of T7‐MNTs was 91.3 ± 4.9 nm, which was greater than that of MNP@SiO_2_ (68.1 ± 5.9 nm) (Figure S1, Supporting Information).

To measure the shielding effect of silica shell coating, the degree of iron release was further detected by 1, 10‐phenanthroline, which is a hetero‐tricyclic nitrogen containing compound to react with Fe^2+^ and form stable complexes with a maximum absorption at 510 nm. As shown in Figure 1f, in the acidic conditions at pH 4.5 or pH 5.5, the bare MNP groups showed a significant absorption at 510 nm. For the T7‐MNTs group, there was no such absorption both in the acidic and normal physiological conditions (pH 7.4). This result suggested that the silica shell could keep the iron oxide nanoparticle core intact, thereby preventing the iron release from T7‐MNTs in the acidic environment. The saturation magnetization value of T7‐MNTs was 84.97 emu/g (Figure 1g), which ensured that T7‐MNTs could readily respond to the magnetic field. Furthermore, when exposed to the 0.5 Hz MF, T7‐MNTs could assemble into rod‐like swarms and rotate synchronously with the external magnetic field (Figure 1h; Video S1, Supporting Information), indicating that the T7‐MNTs could serve as effective transducers for the MF. According to literature reported, destruction of the attraction between lipid membrane and membrane protein required a force from 30 to 50 pN.^[^ ^19^ ^]^ The forces generated by rotating rod‐like T7‐MNTs swarms with 375 nm length under MF of low frequency (40 mT, 0.5 Hz) were simulated to be 36.4 pN (Figure S2, Supporting Information), which could meet the requirement to mechanically damage the lipid membrane.

### 2.2 Targeting Ability of T7‐MNTs in Cancer Cells

**Figure 2:**
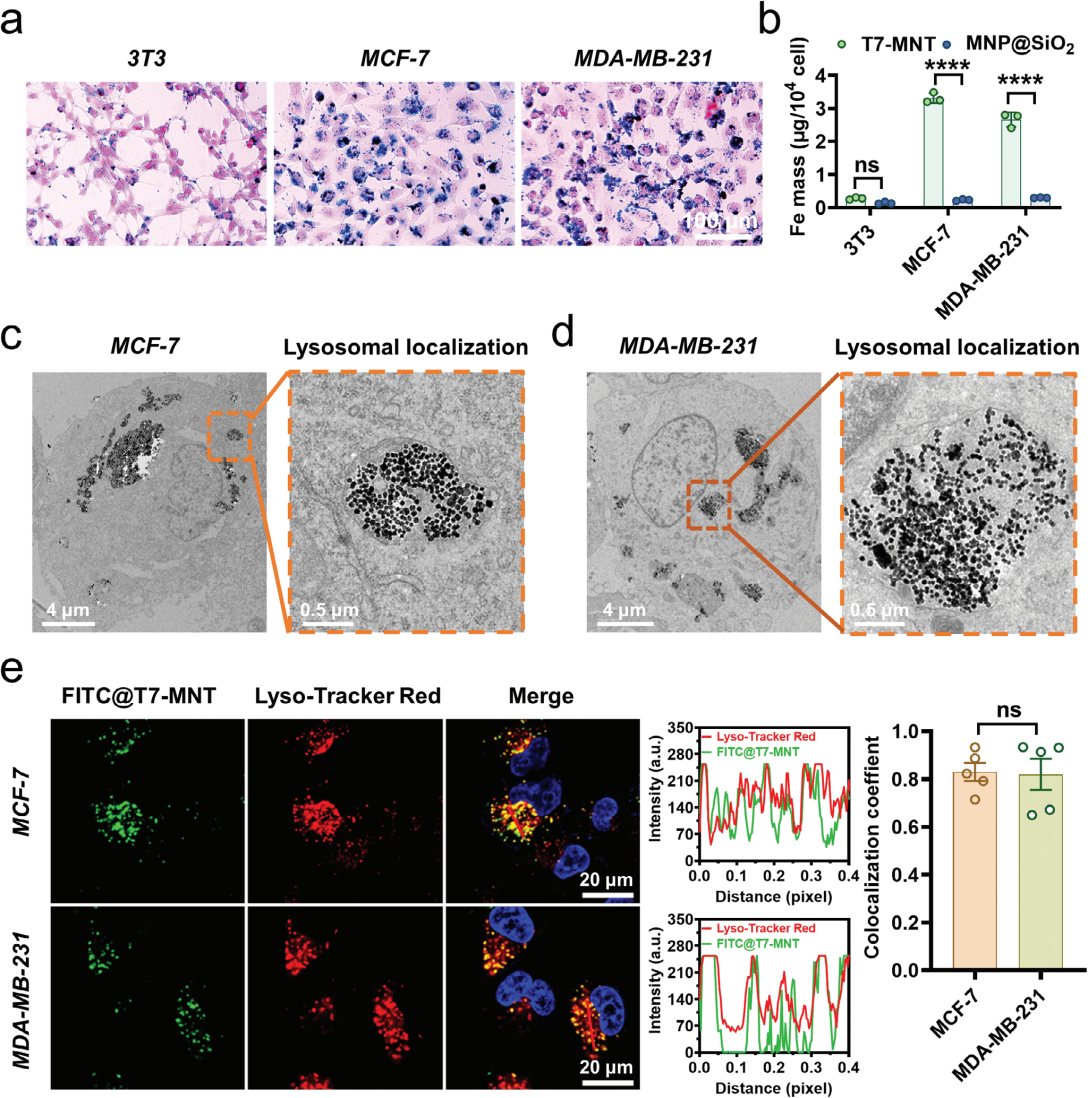
Lysosome targeting ability of T7‐MNTs in cancer cells. a) Prussian blue staining of T7‐MNTs co‐incubated with three different cell lines (3T3, MCF‐7, and MDA‐MB‐231 cells) for 24 h (scale bar: 100 µm). b) The internalized Fe mass in 3T3, MCF‐7, and MDA‐MB‐231 cells after co‐incubation with T7‐MNTs and MNP@SiO_2_, respectively (*n* = 3, ****p < 0.0001, ns: no significance). c,d) Bio‐TEM images of T7‐MNTs located in lysosomes inside MCF‐7 c) and MDA‐MB‐231 cells d) (scale bar: 4 µm, 0.5 µm). e) Confocal fluorescence images and colocalization coefficient of FITC@T7‐MNTs with lysosomes in MCF‐7 and MDA‐MB‐231 cells (scale bar: 20 µm). The representative line profiles of FITC@T7‐MNTs and Lyso‐Tracker Red (red line in images) were measured by Image J (*n* = 5, ns: no significance).

To verify the targeting capacity of T7‐MNTs to breast cancer cells, two cancer cell lines (MCF‐7 and MDA‐MB‐231 cells) and one normal fibroblast cell line (3T3 cells) were performed for transferrin receptors expression study. As shown in Figure S3 (Supporting Information), MCF‐7 and MDA‐MB‐231 cells possessed an overexpression of transferrin receptors on the membrane when compared to 3T3 cells, due to the high‐iron dependency growth of breast cancer cells.^[^ ^20^ ^]^ The cellular uptake of T7‐MNTs in the three cell lines was further investigated. As shown in **Figure** **2**a, T7‐MNTs could target MCF‐7 and MDA‐MB‐231 cells efficiently. Meanwhile, after 24 h co‐incubation, the cellular uptake of T7‐MNTs in MCF‐7 cells (3.3 ± 15.1 µg per 10^4^ cells) and MDA‐MB‐231 cells (2.7 ± 21.6 µg per 10^4^ cells) was 14.3 and 8.9 times higher than that of MNP@SiO_2_ (0.2 ± 2.3 µg per 10^4^ cells and 0.3 ± 1.0 µg per 10^4^ cells) (Figure 2b; Figure S4a, Supporting Information), suggesting that the modification of T7 peptides could effectively enhance the internalization of T7‐MNTs to breast cancer cells. Moreover, the cytotoxicity of T7‐MNTs co‐incubated with the breast cancer cells (MCF‐7 and MDA‐MB‐231 cells) was determined. After 7 days co‐culture with breast cancer cells, T7‐MNTs showed a good biocompatibility with a concentration of 50 µg mL^−1^. The cell viabilities were 94% and 97% in MCF‐7 and MDA‐MB‐231, respectively (Figure S4b,c, Supporting Information). As shown in bio‐TEM images (Figure 2c,d), T7‐MNTs mainly located in the lysosomes of MCF‐7 and MDA‐MB‐231 cells. Furthermore, confocal images showed that most green fluorescence signals of T7‐MNTs colocalized well with red fluorescence signals of lysosomes, with average colocalization coefficients (tMg, Manders’ overlap coefficient for green signals in thresholded image) of 0.83 and 0.82, respectively (Figure 2e). However, there was no obvious colocalization of T7‐MNTs with mitochondria, endoplasmic reticulum, and F‐actin (Figure S5, Supporting Information), indicating that the good lysosome‐targeting property of T7‐MNTs. The transferrin receptor‐mediated endocytosis pathway is essential for the lysosome‐targeted ability of T7‐MNTs. Specifically, extracellular transferrin could bind to the transferrin receptor to form the transferrin receptor/transferrin (TFR/TF‐(Fe^3+^)_2_) complex. The TFR/TF‐(Fe^3+^)_2_ complexes can be subsequently wrapped in a clathrin‐coated pit to undergo endocytosis, and then eventually transported to the lysosomes.^[^ ^21^ ^]^ To further confirm the endocytosis pathway of T7‐MNTs, clathrin‐dependent endocytosis pathway inhibition experiment and receptor competitive experiment were performed. As shown in Figure S6 (Supporting Information), compared with the control group, the treatment of chlorpromazine (an inhibitor of clathrin‐dependent endocytosis pathway) displayed a significant inhibition in cellular uptake of T7‐MNTs both in MCF‐7 and MDA‐MB‐231 cells. Notably, as indicated by the yellow spots in Figure S6 (Supporting Information), T7‐MNTs did not show obvious colocalization with lysosomes after chlorpromazine treatment, indicating that the internalization and lysosomal colocalization of T7‐MNTs were clathrin‐dependent endocytosis. With the presence of excess anti‐transferrin receptor antibodies, the cellular uptake and lysosomal colocalization of T7‐MNTs were significantly decreased. These results demonstrated that T7‐MNTs could efficiently target lysosomes via the transferrin receptor‐mediated endocytosis. Above all, the high internalization and lysosomal colocalization efficiency of T7‐MNTs established a foundation for the mechanical torques to locally act on the lysosomes.

### 2.3 Disruption of Lysosomes and Endogenous Fe^2+^ Pool Outbreak via T7‐MNTs

**Figure 3:**
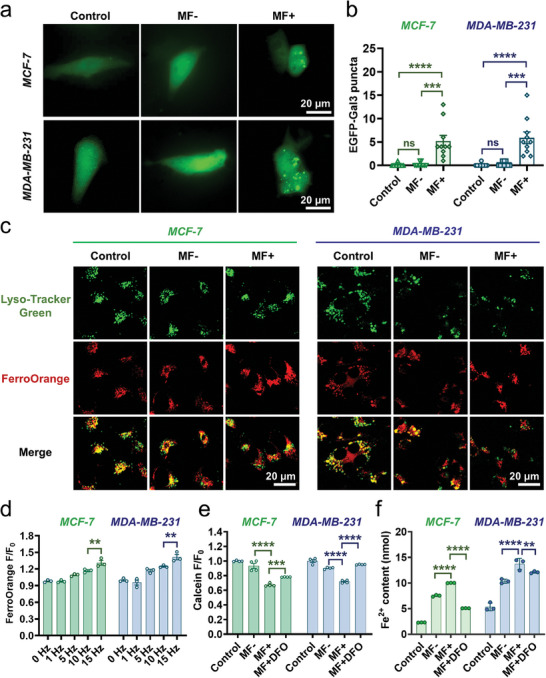
Disruption of lysosomes and endogenous Fe^2+^ pool outbreak via T7‐MNTs. a) Representative fluorescence images of lysosomal membrane permeabilization in MCF‐7 and MDA‐MB‐231 cells (scale bar: 20 µm). b) Quantitative analysis of EGFP‐Gal3 fluorescent puncta in MCF‐7 and MDA‐MB‐231 cells by Image J (*n* = 10, ****p < 0.0001, ***p < 0.001, ns: no significance). c) Confocal images of intracellular iron distribution in MCF‐7 and MDA‐MB‐231 cells with or without MF treatment, untreated cells were used as the control group (scale bar: 20 µm). d) Quantitation of FerroOrange fluorescence in MCF‐7 and MDA‐MB‐231 cells after different MF frequency conditions (*n* = 3, **p < 0.01). e) Quantitation of Calcein quenching after different conditions (*n* = 4, ****p < 0.0001, ***p < 0.001). f) Intracellular Fe^2+^ level in MCF‐7 and MDA‐MB‐231 cells treated with different conditions (*n* = 3, ****p < 0.0001, **p < 0.01).

As shown in bio‐TEM images, the T7‐MNTs localized in lysosomes could also assemble into rod‐like swarms in MCF‐7 and MDA‐MB‐231 cells under MF (Figure S7, Supporting Information). According to our previous work,^[^ ^16a^ ^]^ the rod‐like T7‐MNTs swarms could synchronously rotate under the MF, further generating mechanical torques to damage the lysosomal membrane by inducing fluidic vortex. Destruction of lysosomal membrane can induce lysosomal membrane permeabilization (LMP), which could be evaluated by quantifying the EGFP‐galectin‐3 (EGFP‐gal3) punctum. When the LMP increased, EGFP‐galectin‐3 could bind with the β‐galactoside‐rich lysosomal glycocalyx and form fluorescent punctum.^[^ ^22^ ^]^ As shown in **Figure** **3**a,b, the T7‐MNTs with MF treatment group (MF+) exhibited the most fluorescent punctum (5.2 ± 3.9 per MCF‐7 cell and 5.9 ± 4.0 per MDA‐MB‐231 cell) in comparison with the material group (MF‐, 0.2 ± 0.4 per MCF‐7 cell and 0.3 ± 0.5 per MDA‐MB‐231 cell) and cells group (control, 0.1 ± 0.3 per MCF‐7 cell and MDA‐MB‐231 cell), suggesting the significant effect of T7‐MNTs on inducing LMP both in MCF‐7 and MDA‐MB‐231 cells. Subsequently, an acidotrophic pH indicator (LysoSensor Yellow/Blue DND‐160), which exhibited predominantly yellow fluorescence in acidic conditions and blue fluorescence in neutral conditions, was used to examine the integrity of lysosomes. As shown in Figure S8 (Supporting Information), the ratio value of yellow/blue fluorescence intensity of the MF+ group (6.65 ± 0.06 in MCF‐7 cells and 5.38 ± 0.55 in MDA‐MB‐231 cells) was lower than that of the MF‐ group (8.69 ± 0.05 in MCF‐7 cells and 7.66 ± 0.02 in MDA‐MB‐231 cells), demonstrating that lysosomal pH decreased after MF treatments which was related to the rupture of the lysosomal membrane.

Moreover, the increment of LMP can promote the leakage of lysosomal contents, especially lysosomal iron into the cytoplasm. Subsequently, the intracellular Fe^2+^ and lysosomes were stained with the ferrous iron imaging probe FerroOrange and Lyso‐Tracker Green, respectively. As seen in Figure 3c, after MCF‐7 and MDA‐MB‐231 cells were treated with T7‐MNTs, more fluorescence signals of FerroOrange (red) appeared in the cytoplasm than lysosomes. In contrast, the fluorescence signals of FerroOrange in the control group and MF‐ group mostly overlapped with the fluorescence signals of lysosomes (green). The colocalization coefficients (tMr, Manders’ overlap coefficient for red signals in the thresholded image) of MF+ group were 0.31 ± 0.13 of MCF‐7 cells and 0.28 ± 0.07 of MDA‐MB‐231 cells, which were lower than those of the control group (0.80 ± 0.03 of both MCF‐7 and MDA‐MB‐231 cells) and MF‐ groups (0.81 ± 0.08 of MCF‐7 cells and 0.83 ± 0.07 of MDA‐MB‐231 cells) (Figure S9, Supporting Information). The results indicated that the torques generated by T7‐MNTs could release endogenous Fe^2+^ from lysosomes, resulting from its lysosomal membrane disruption capacity. As shown in Figure 3d, the fluorescent intensity of FerroOrange increased along with the frequency of MF (5, 10, and 15 Hz), ensuring the dynamic regulation of the endogenous Fe^2+^ liberation under the MF. Meanwhile, in the 15 Hz group, the highest fluorescence intensity of FerroOrange was observed, suggesting that the 15 Hz induced the torques possessed the best LMP effect. Considering the endogenous Fe^2+^ released from lysosomes could enhance the labile iron pool (LIP) level, the LIP level was quantified by calcein acetoxymethyl ester (Calcein‐AM), which was well retained in the cytoplasm instead of organelles and could be quenched by the chelation with Fe^2+^.^[^ ^23^ ^]^ Compared with that in the control group, the fluorescence intensity of Calcein‐AM in the MF+ group significantly decreased 1.49 times in MCF‐7 cells and 1.39 times in MDA‐MB‐231 cells. Then, with the addition of deferoxamine (DFO), an iron‐chelating agent and ferroptosis inhibitor, the fluorescence intensity of Calcein‐AM remained analogous to the control group, suggesting that the decrease of fluorescence intensity of Calcein‐AM was caused by the chelation of Fe^2+^ (Figure 3e). Furthermore, as shown in Figure 3f, the MF+ group showed an increase in total Fe^2+^ level higher than MF‐ group (p < 0.0001). Similar results were also observed in MDA‐MB‐231 cells. These results proved that the proposed T7‐MNTs could disrupt lysosome structure and dynamically break the endogenous Fe^2+^ pool. Recently, other physical‐responsive inducers based on light, ultrasound and microwave also possessed the ability to cause lysosome dysfunction^[^ ^24^ ^]^ to release loaded irons^[^ ^25^ ^]^ or accelerate the ferritin degradation^[^ ^26^ ^]^ for enhancement of the intracellular iron level. Generally, the precise lysosome‐targeted regulation was often constrained in vivo due to the insufficient control for deep‐seated tumors^[^ ^27^ ^]^ and undesirable side effects.^[^ ^28^ ^]^ To overcome these limitations, the T7‐MNTs could achieve dynamic on‐demand regulation of endogenous Fe^2+^ pool by generating the localized mechanical torquers to trigger the LMP in a long‐lasting and spatiotemporal manner.

### 2.4 T7‐MNTs‐Induced Oxidative Damage and Antioxidant Defense Imbalance for Ferroptosis

**Figure 4:**
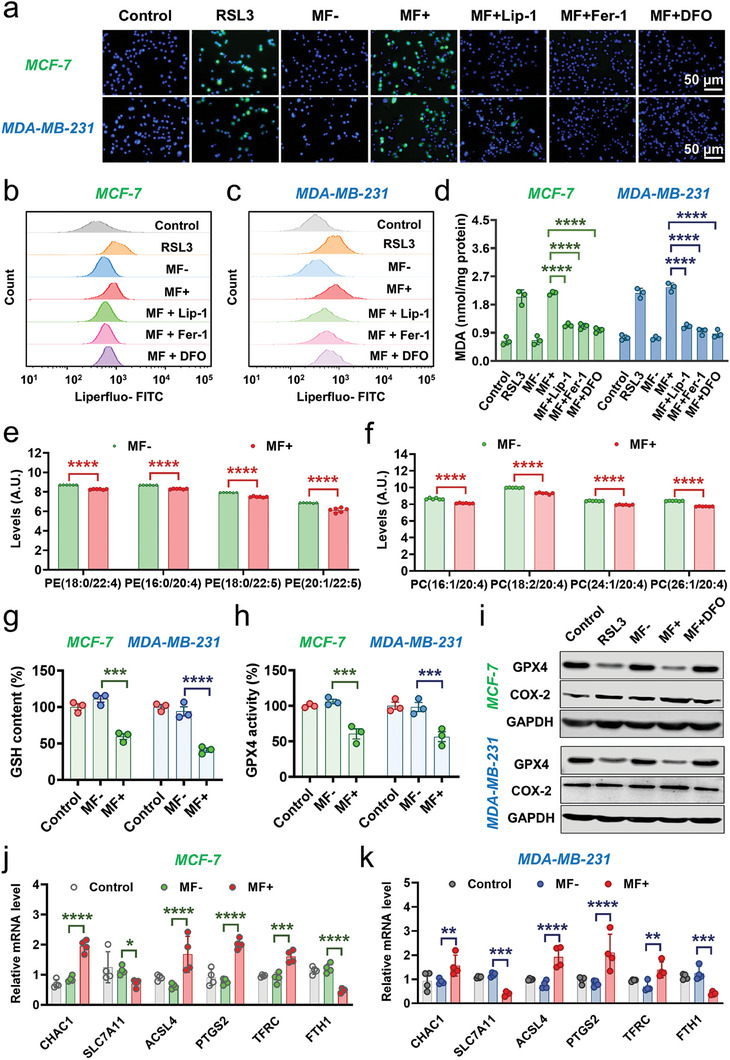
Iron‐driven oxidative damage and antioxidant defense imbalance via T7‐MNTs. a) Fluorescence images of intracellular ROS level in MCF‐7 and MDA‐MB‐231 cells measured by DCFH‐DA staining. RSL3 group was treated with RSL3 (20 µM) for 24 h. The “MF+” group was treated with T7‐MNTs and followed by MF application (260 mT, 15 Hz, 30 min). “MF+Lip‐1″, “MF+Fer‐1″, and “MF+DFO” groups were incubated with Lip‐1 (1 µm), Fer‐1 (5 µm), and DFO (50 µm) for 6 h before MF application. The cells incubated with T7‐MNTs were used as the “MF‐” group, and untreated cells were used as the control group. (scale bar: 50 µm). b,c) Flow cytometry results of LPO level in MCF‐7 (b) and MDA‐MB‐231 cells (c) after different treatments. d) MDA content of MCF‐7 and MDA‐MB‐231 cells with various conditions (*n* = 3, ****p < 0.0001). e) Levels of PEs in the MF‐ and MF+ groups (*n* = 6, ****p < 0.0001). f) Levels of PCs in the MF‐ and MF+ groups (*n* = 6, ****p < 0.0001). g) Intracellular GSH level of MCF‐7 and MDA‐MB‐231 cells (*n* = 3, ****p < 0.0001, ***p < 0.001). h) Intracellular GPX4 activity of MCF‐7 and MDA‐MB‐231 cells (*n* = 3, ***p < 0.001). i) Western blotting assay of the GPX4 protein and COX‐2 protein expression after different treatments in MCF‐7 and MDA‐MB‐231 cells, respectively. j–k) The mRNA expression level of CHAC1, SLC7A11, ACSL4, PTGS2, TFRC, and FTH1 with different treatment in MCF‐7 j) and MDA‐MB‐231 cells k). (*n* = 4, ****p < 0.0001, ***p < 0.001, **p < 0.01, *p < 0.05).

Considering the released endogenous Fe^2+^ induced by T7‐MNTs was redox‐active, the ROS level was measured after T7‐MNTs treatment via the 2′,7′‐dichlorofluorescein diacetate (DCFH‐DA) staining. As shown in **Figure** **4**a, the stronger green fluorescence signals were observed both in the MF+ group and RSL3 group, indicating that the MF+ group exhibited remarkable enhancement of ROS production. However, the green fluorescence signals could be barely observed in the liproxstatin‐1 (Lip‐1, a phospholipid radical‐trapping agent), ferrostatin‐1 (Fer‐1, a phospholipid peroxyl radical scavenger), and DFO groups, indicating significant downregulation of the cellular ROS level of those groups. Combining these results together, it was confirmed that the release of lysosomal Fe^2+^ induced by T7‐MNTs could improve the Fenton reaction and further increase intracellular ROS levels. Advancedly, the strong ROS production could further amplify LMP^[^ ^29^ ^]^ to accelerate the endogenous Fe^2+^ liberation from lysosome to enhance the Fenton reaction then creating an endogenous Fe^2+^ pool outbreak positive feedback circuit. Owing to the redox‐active endogenous Fe^2+^ released from lysosomes could promote the generation of oxidative radicals and accelerate lipid peroxidation, the intracellular level of lipid peroxides (LPO) was investigated through Liperfluo staining via flow cytometry. As shown in Figure 4b,c, the fluorescence intensity of the Liperfluo in the MF+ group was higher than that of other control groups, which was consistent with the RSL3 treatment group, indicating that T7‐MNTs treatment provoked a high LPO level. Similarly, MCF‐7 and MDA‐MB‐231 cells groups receiving Lip‐1, Fer‐1, and DFO treatments showed lower accumulation of LPO levels. Malondialdehyde (MDA), the toxic by‐product of LPO was further evaluated. As shown in Figure 4d, the levels of MDA were dramatically increased after T7‐MNTs treatment, which were approximated to that of the RSL3 group and higher than that of the inhibitor (Lip‐1, Fer‐1, DFO) treated groups, suggesting that upregulated ROS levels could boost the level of LPO in the two cell lines. Moreover, the lipid metabolomics analysis was performed to study the changes of lipids after T7‐MNTs treatment. Many studies have reported that polyunsaturated fatty acid (PUFA)‐containing lipids, including phosphatidylethanolamine (PE) and phosphatidylcholine (PC), are decreased in lipid peroxidation.^[^ ^30^ ^]^ As shown in Figure S10a (Supporting Information), principal component analysis (PCA) result revealed a clear separation between MF‐ and MF+ group, suggesting the significant change of the lipids expression in response to T7‐MNTs treatment. Strikingly, 565 of lipid metabolites (335 downregulated and 230 upregulated) exhibited the most significant changes upon T7‐MNTs treatment (Figure S10b, Supporting Information). Moreover, the levels of several PUFA‐containing PE/PC decreased in T7‐MNTs‐treated cells, indicating that the T7‐MNTs could facilitate lipid peroxidation for arousing ferroptosis (Figure 4e,f).

As an important intracellular antioxidant enzyme, glutathione peroxidase 4 (GPX4) can directly reduce LPO to hydroxy phospholipids (PL) and restrain lipid peroxidation.^[^ ^31^ ^]^ Moreover, the intracellular glutathione (GSH) level is crucial for maintaining GPX4 activity to reduce lipid hydroperoxides.^[^ ^32^ ^]^ Then, GSH and GPX4 performance were further evaluated. As shown in Figure 4g, compared with the MF‐ group, the MF+ group in MCF‐7 cells exhibited a decreased GSH level (59.34 ± 6.87% of that in the control group), and the MF+ group in MDA‐MB‐231 cells exhibited a higher decreased GSH level (40.83 ± 4.91%), indicating that T7‐MNTs could trigger GSH depletion. Additionally, lower GPX4 activity was observed in the MF+ group (60.41 ± 12.34% of MCF‐7 cells and 56.56 ± 12.05% of MDA‐MB‐231 cells) (Figure 4h). Western blot experiment also confirmed that the expression of GPX4 was apparently downregulated compared to MF‐ group, which was attributed to the depletion of GSH (Figure 4i). However, the downregulation of GPX4 was reversed with the addition of DFO. Theoretically, T7‐MNTs mediated the enhancement of LIP could generate high levels of free radicals via Fenton reaction, and the excessive free radicals could oxidize phospholipids (PL) to generate phospholipid peroxyl radical (LOO•) for overproduction of LPO and result in excess intracellular ROS stress,^[^ ^33^ ^]^ which could deplete intracellular GSH and indirectly lead to the inactivation of GPX4.

To further validate the effect of T7‐MNT‐induced oxidative damage and antioxidant defense imbalance for ferroptosis, the expression levels of representative ferroptosis‐related genes was detected by the real‐time quantitative polymerase chain reaction (RT‐qPCR) analysis. These included cation transport regulator homolog 1 (CHAC1, an enzyme to degrade intracellular GSH into 5‐OH and Cys–Gly), amino acid antiporter solute carrier family 7 member 11 (SLC7A11, a key regulator of GSH synthesis), prostaglandin‐endoperoxide synthase 2 (PTGS2, the key enzyme in prostaglandin biosynthesis), acyl‐CoA synthetase long‐chain family member 4 (ACSL4, an enzyme involved in fatty acid metabolism), iron importers transferrin receptor protein (TFRC, a major regulator of iron import) and ferritin heavy chain 1 (FTH1). As shown in Figure 4j and Figure 4k, the upregulated mRNA levels of CHAC1 and the downregulated mRNA level of SLC7A11 by T7‐MNTs treatment indicated that the intracellular GSH levels were depleted. Additionally, western blot experiments also confirmed that the T7‐MNTs treatment induced the most significant downregulation of SLC7A11 (Figure S11, Supporting Information). Since the expression of PTGS2 and ACSL4 increase with the promotion of lipid peroxidation‐dependent ferroptosis,^[^ ^34^ ^]^ the upregulation of the expression of both genes in the MF+ group demonstrated that ferroptosis could be triggered efficiently by the T7‐MNTs treatment. As the upstream oxidase of the PTGS2 synthesis, the cyclooxygenase 2 (COX‐2) was also upregulated after T7‐MNTs treatment (Figure 4i). The ferritin that stored endogenous Fe^2+^, showed most downregulated expression in MF+ group (Figure S11, Supporting Information). Additionally, the promotion of the expression of TFRC and the decrease of the expression of FTH1 after the T7‐MNTs treatment, suggested the active uptake of extracellular iron and the degradation of ferritin, together leading to intracellular iron overload for ferroptosis activation. Collectively, these results demonstrated that T7‐MNTs could facilitate lipid peroxidation for arousing ferroptosis by enhancing the Fe^2+^‐driven oxidative damage and malfunctioning the intracellular antioxidant defense.

### 2.5 Frequency‐ and Time‐Dependent Ferroptosis via T7‐MNTs

**Figure 5:**
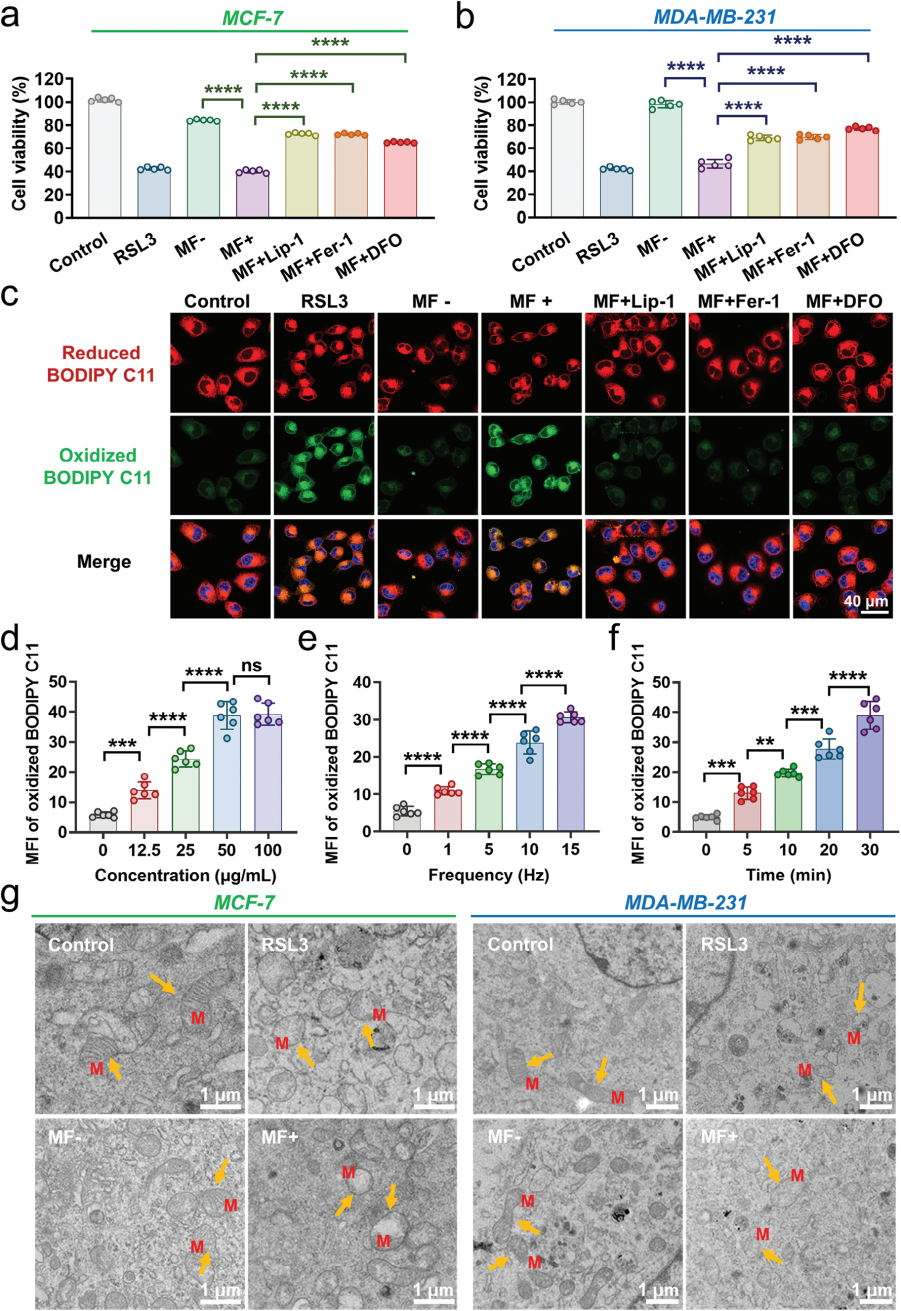
T7‐MNT‐mediated ferroptosis in cancer cells. a,b) Cell viability of MCF‐7 a) and MDA‐MB‐231 cells b) with different treatments (*n* = 5, ****p < 0.0001). The “MF+” group was treated with MF (260 mT, 15 Hz) for 30 min. “MF+Lip‐1″, “MF+Fer‐1″, and “MF+DFO” groups were respectively pre‐treated with Lip‐1 (1 µm), Fer‐1 (5 µm), and DFO (50 µm) for 6 h before MF. The cells incubated with T7‐MNTs were used as the “MF‐” group, and untreated cells were used as the control group. c) Confocal images of lipid peroxidation in MCF‐7 cells after different treatments. 20 µm of RSL3 was used as a positive control (scale bar: 40 µm). d) Quantitative analysis of oxidized BODIPY C11 in MCF‐7 cells treated with different concentrations of T7‐MNTs (Fe = 0, 12.5, 25, 50, 100 µg mL^−1^). The frequency of MF was 15 Hz and the treatment time was 30 min. e) Quantitative analysis of oxidized BODIPY C11 in MCF‐7 cells treated with different frequencies of MF. The concentration of T7‐MNTs was 50 µg mL^−1^ and the treatment time of MF application was 30 min. f) Quantitative analysis of oxidized BODIPY C11 in MCF‐7 cells treated with different treatment periods. The concentration of T7‐MNTs was 50 µg mL^−1^, and the frequency of the MF was 15 Hz. (*n* = 6, ****p < 0.0001, ***p < 0.001, **p < 0.01, ns: no significance). g) Typical bio‐TEM images of MCF‐7 and MDA‐MB‐231 cells with different treatments. Yellow arrows noted the representative morphology of mitochondria. M represented mitochondria (scale bar: 1 µm).

To verify the ferroptosis occurrence induced by T7‐MNTs treatment, ferroptosis inhibitors were utilized to detect the cytotoxicity of T7‐MNTs treated with MCF‐7 and MDA‐MB‐231 cells under MF. As shown in **Figure** **5**a, the viability of MCF‐7 cells treated with free T7‐MNTs could remain over 84% in MF‐ group, whereas T7‐MNTs induced significant cell death of 60% under MF treatment (MF+), which was similar to the results of RSL3‐treated cells. Notably, when using ferroptosis inhibitors with MF treatment, such as Lip‐1, Fer‐1 and DFO, the cell viability of MCF‐7 cells induced by T7‐MNTs raised to over 65%, respectively. These results suggested that ferroptosis was the prominent form of cell death during T7‐MNTs‐coupled MF treatment. Similar results were confirmed in the MDA‐MB‐231 cell lines (Figure 5b). T7‐MNTs also stimulated apparent ferroptosis with MDA‐MB‐231 cell death of 53%. Moreover, T7‐MNTs induced obvious cell death of A549 cells (38%) and HepG2 cells (54%) under MF treatment (Figure S12, Supporting Information), whereas T7‐MNTs treatment exhibited neglectable cytotoxicity to 3T3 cells (cell viability of 91.26%) (Figure S13, Supporting Information). These results suggested that the T7‐MNTs with the T7 peptide modification ensured the specific recognition and target cancer cells, further realizing the selectivity of T7‐MNTs‐mediated ferroptosis for in vivo studies.

C11‐BODIPY581/591, as a specific lipid peroxidation probe, has been used for further determination of ferroptosis. Its fluorescence signals can change from red (reduced) to green (oxidized) upon the reaction with lipid peroxides in living cells. As shown in Figure 5c and Figure S14 (Supporting Information), compared to the control group, stronger green fluorescence signals were observed in RSL3 group, indicating an up‐regulation of lipid peroxidation and enhancement of ferroptosis. Consistently, MF+ group showed significant oxidized fluorescence signals (green), while ferroptosis inhibitors (Lip‐1, Fer‐1, and DFO) treated groups showed weak signals (green). These results further determined that ferroptosis was successfully induced by the T7‐MNTs‐generated torque under a programmed rotating magnetic field. Next, to investigate ferroptosis effect manipulated by the programmed strategy, we conducted a series of experiments by varying the concentration of T7‐MNTs, the frequency of MF, and the MF exposure time. As shown in Figure 5d and Figure S15 (Supporting Information), the fluorescence intensity of oxidative BODIPY C11 in these cancer cells also showed an increased intensity with the T7‐MNTs concentration, suggesting that the enhancement of ferroptosis occurrence exhibited the concentration‐dependent manner. Thereinto, MCF‐7 cells treated with 50 µg mL^−1^ T7‐MNTs showed the most excellent ferroptosis effect. With the increase of MF frequency from 0 to 15 Hz, the fluorescence intensity of oxidative BODIPY C11 in MCF‐7 and MDA‐MB‐231 cells gradually increased, which was similar to the tendency of MF exposure time extension from 0 to 30 min (Figure 5e,f; Figures S16 and S17, Supporting Information). The above results indicated that the regulation of ferroptosis via T7‐MNTs was MF frequency‐ and time‐dependent. In addition, the cell morphology features during ferroptosis were observed due to the bio‐TEM images (Figure 5g). T7‐MNTs‐treated cells exhibited obvious swelling of mitochondria with vanish cristae, which was consistent with the ferroptosis features of cancer cells induced by RSL3. All the results suggested that the magneto‐mechanical regulation by T7‐MNTs evoked MF frequency‐ and time‐dependent ferroptosis significantly. Although substantial research efforts have developed various magnetism‐responsive nanomaterials to perturb the organelles^[^ ^16^, ^35^ ^]^ to trigger cancer cell death, the mechanism of the cell death was mainly focused on the apoptosis.^[^ ^36^ ^]^ The T7‐MNTs as the magneto‐mechanical inducer offered an insight of magneto‐mechanical regulation to trigger the outbreak of endogenous labile iron pool for the long‐acting and selective ferroptosis in cancer therapy.

### 2.6 Mechanical Targeted Ferrotherapy In Vivo via T7‐MNTs

**Figure 6:**
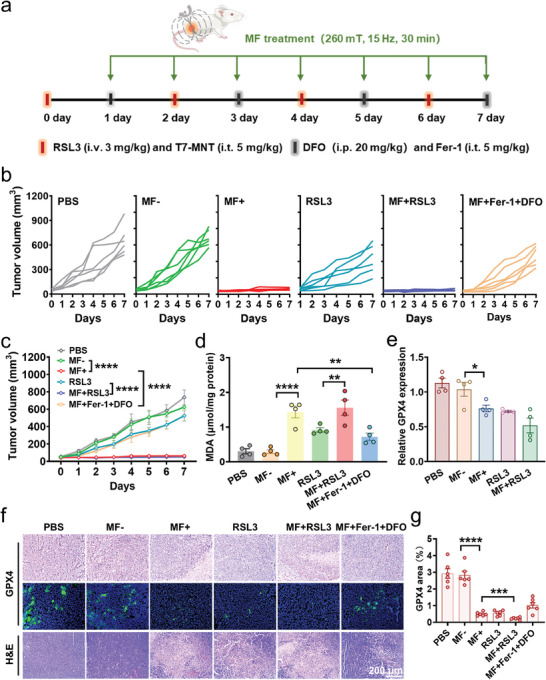
In vivo antitumor effects of T7‐MNTs on MDA‐MB‐231 tumor‐bearing mice. a) Schematic diagram of the treatment process of MDA‐MB‐231‐bearing mice. T7‐MNTs (5 mg kg^−1^, i.t.) and RSL3 (3 mg kg^−1^, i.v.) were injected on days 0, 2, 4, and 6 for four times. Fer‐1 (5 mg kg^−1^, i.t.) and DFO (20 mg kg^−1^, i.p.) were injected on days 1, 3, 5, and 7 for four times. The MF application (with therapeutic parameters of 260 mT, 15 Hz) was performed for 7 days with 30 min per day. b) Tumor growth curves of each mouse in six groups. c) Tumor volume of mice recorded daily for 7 days from different treatment groups (*n* = 4, ****p < 0.0001). d) MDA content of tumor tissue in different treatment groups (*n* = 4, ****p < 0.0001, **p < 0.01). e) The mRNA expression level of GPX4 after different treatments (*n* = 4, *p < 0.05). f) Immunohistochemical analysis and immunofluorescent analysis of GPX4 and H&E staining of tumor slices in different treatment groups (scale bar: 200 µm). g) Statistical summary of the GPX4 area in the tumors (*n* = 6, each dot represents an analyzed tumor section, ****p < 0.0001, ***p < 0.001).

Encouraged by these excellent ferroptosis therapeutic effects in vitro, the mechanical targeted ferrotherapy had been applied to the MDA‐MB‐231 tumor‐bearing mice. As exhibited in the illustrated treatment procedure (**Figure** **6**a), a total of 36 MDA‐MB‐231 tumor‐bearing mice were randomly separated into six groups (PBS, RSL3, MF‐, MF+, MF+RSL3, and MF+Fer‐1+DFO, n = 6 for each group). The PBS and MF‐ (i.t. injected with 5 mg kg^−1^ T7‐MNTs but without MF treatment) groups were used as the control. The RSL3 group (i.v. injected with 3 mg kg^−1^ RSL3) was used as a ferroptosis positive group. The MF+ group was i.t. injected with 5 mg kg^−1^ T7‐MNTs and then received MF treatment of 30 min every day for 7 days. To validate the ferroptosis occurrence, the two ferroptosis inhibitors (Fer‐1 and DFO) were additionally injected every other day following the T7‐MNTs treatment. During the treatment, tumor volume was measured daily. The tumor growth curves of the six groups were displayed in Figure 6b,c. Compared to the PBS and MF‐ groups, MF+ group showed remarkable inhibition of tumor growth, indicating that T7‐MNTs possessed excellent antitumor efficiency due to their ability to induce the outbreak of endogenous Fe^2+^ pool. Notably, even though RSL3 showed obvious cytotoxicity in vitro, the in vivo antitumor effect of RSL3 group was poorer than that of MF+ group, suggesting that the treatment of RSL3 in vivo still faced challenges such as fast drug metabolism and short‐acting efficacy. Furthermore, combined with RSL3 group, the MF+RSL3 group showed enhanced antitumor effects. The result indicated that the combination treatment of RSL3 and T7‐MNTs could achieve a synergistic antitumor effect to improve the ferroptosis‐induced efficiency triggered by RSL3. It could be attributed to the accumulation of intracellular labile Fe^2+^ induced by T7‐MNTs to enhance the vulnerability of tumor cells to ferroptosis. Strikingly, the antitumor effect was greatly inhibited by the treatments of Fer‐1 and DFO, suggesting that the effective ferroptosis regulation was induced via the magnetic torques generated by T7‐MNTs under MF. To evaluate the long‐term antitumor efficacy of the T7‐MNTs treatment, an additional observation period was added in the experiment (Figure S18a, Supporting Information). During the treatment and observation period, tumor volume and body weight were measured every 2 days. Notably, a sustained inhibition of tumor growth in T7‐MNTs‐treated groups (MF+ and MF+RSL3) was exhibited (Figure S18b, Supporting Information). The tumor weight of the MF+ group (0.346 ± 0.03 g) was significantly lower than that of the MF‐ group (0.868 ± 0.17 g), similar to the results of MF+RSL3 group (0.303 ± 0.01 g) (Figure S18c, Supporting Information). The digital photographs of tumors of each group were displayed in Figure S18d (Supporting Information). Additionally, the antitumor effect of T7‐MNTs was inhibited significantly after the simultaneous treatments of Fer‐1 and DFO, compared to the antitumor effects inhibited by the individual treatment of Fer‐1 or DFO. The corresponding results further suggested that T7‐MNTs treatment could trigger ferroptosis effectively. All the treated mice showed slight body weight changes during the treatment, and all the major organs had well‐organized cellular structures, indicating that T7‐MNTs‐coupled MF treatment showed negligible negative impacts on the mice (Figure S18e,f, Supporting Information). Collectively, the above tumor inhibition induced by MF treatment revealed that T7‐MNTs could serve as a long‐acting physical inducer and play a long‐term therapeutic effect with a suitable dose.

The in vivo MDA accumulation and GPX4 inactivation performances of T7‐MNTs‐induced ferroptosis were further evaluated. After 7 days treatment, tumor tissues were collected from euthanized mice for subsequent analysis. As seen in Figure 6d, the MDA level of the MF+ group was 4.9 times higher than that of MF‐ group, evidencing high lipid peroxidation levels in tumor sites by T7‐MNTs after MF treatment. Notably, the MF+RSL3 group showed higher MDA levels than the RSL3 group and MF+ group, further confirming that T7‐MNTs could cooperate with RSL3 to robustly enhance the intracellular LPO level for activating ferroptosis. Furthermore, the mRNA expression levels of GPX4 of tumor tissues were downregulated in the MF+ and MF+RSL3 groups compared to other groups (Figure 6e). As shown in Figure 6f,g, the expression of GPX4 exhibited noticeable downregulation in T7‐MNTs‐treated groups, which was consistent with the mRNA results. Moreover, the MF+ and MF+RSL3 groups exhibited abundant tumor cell death by the hematoxylin and eosin (H&E) staining (Figure 6f), revealing the capability of T7‐MNTs in GPX4 inhibition and ferroptosis induction. Taken together, these results proved that T7‐MNTs could play a long‐term remarkable therapeutic effect under the programmed MF stimulation. Additionally, with the advantages of MF regulation, the magneto‐mechanical approach could effectively enhance ferrotherapy efficacy by comminating with RSL3. Considering the vulnerability of ferroptosis in the drug‐resistant cancer cells, the T7‐MNTs showed huge potential for achieving the selective cancer cell destruction and overcoming chemo‐resistance for deep‐seated tumors.

## 3 Conclusion

In summary, a lysosome‐targeted magnetic nanotorquer was designed to mechanically trigger ferroptosis in a spatiotemporal, programmable, and long‐acting fashion for cancer treatment. By selectively targeting the transferrin receptors on cancer cells, T7‐MNTs accumulated in lysosomes. Under the programmed rotating magnetic field, the proposed T7‐MNTs could generate torques to precisely disrupt the lysosomal membrane, accompanied by the release of endogenous Fe^2+^. The magneto‐mechanical strategy could promote the Fenton reaction and induce the formation of high ROS. Moreover, the depletion of GSH and deactivation of GPX4 demonstrated that the torques generated by T7‐MNTs inside of the cellular lysosomes could cause the imbalance of oxidative damage and antioxidant defense. Finally, the ferroptosis occurrence could be programmed by adjusting the magnetic field frequency, T7‐MNT concentrations, as well as treatment time. Notably, both in vitro and in vivo studies proved that T7‐MNTs could initiate targeted ferroptosis and achieve enhanced efficacy for cancer treatment. To the best of our knowledge, it is a proof‐of‐concept study to demonstrate the nanomaterial‐based mechanical inducer to trigger the endogenous labile iron pool for targeted ferroptosis. Although numerous small molecules have been developed to target ferroptosis for cancer treatment, their clinical development is still limited by the low selectivity and undesired side effect. T7‐MNTs showed the targeting ability to the lysosomes in the breast cancer cells, which offer a physical and biological platform to collaboratively achieve GPX4 inhibition and ferroptosis induction in vivo. We anticipate this dynamic targeted strategy could be readily integrated with current ferroptosis inducers to achieve enhanced efficacy for clinical cancer treatment. Our work is an example of offering a new perspective and approach for controllable ferroptosis and drive the clinical translation of ferroptosis‐based cancer therapy.

## 4 Experimental Section

### Synthesis of T7‐MNTs

60 nm zinc doped cube‐shaped MNPs were synthesized according to the previous work.^[^ ^17^ ^]^ To prevent the leakage of exogenous iron, the surface of MNPs were coated with SiO_2_ shell. In the reaction system, 7.8 mL of Igepal CO‐520 and 12 mg of MNPs were dispersed in 120 mL of cyclohexane. Then, 1.05 mL of ammonium hydroxide and 300 µL of TEOS were added into solution under sonicating. Next, the mixture was stirred at 480 rpm for 48 h, 60 µL of silane‐PEG2000‐NHS was added, following by keeping stirring for 24 h. Finally, the MNP@SiO_2_ was dispersed in phosphate buffer (pH 8.5). Next, 1 mg of T7 peptide was dissolved in 2 mL of phosphate buffer (pH 8.5) and mixed with 2 mg of MNP@SiO_2_. The mixture was treated by ultrasonic (working time 5 s, interval time 2 s) for 90 min. Afterwards, T7 peptide@MNP@SiO_2_ (T7‐MNT) was washed with ultrapure water three times and dispersed in 15 mL ultrapure water.

### Iron Release Assay

To detect the iron release of T7‐MNTs and bare MNPs, 2 mg of T7‐MNTs and bare MNPs were immersed into 1 mL of PBS at different pH (4.5, 5.5, 7.4) and incubated for 24 h, respectively. The supernatants of all the samples were collected for phenanthroline spectrophotometric analysis by UV−vis spectrophotometer (Cary 60, Agilent).

### Receptor Expression Study

6×10^4^ 3T3, MDA‐MB‐231, and MCF‐7 cells were seeded on each confocal dish (25 mm) and cultured for 24 h, respectively. Then, cells were stained by Hoechst 33 342 for 20 min at 37°C. To promote cell membrane permeability, all groups were fixed with 4% paraformaldehyde for 10 min, and then incubated with PBS containing 0.1% Triton X‐100 for 5 min. Next, cells were blocked by BSA (1%, w/v) for 1 h. Finally, cells were incubated with anti‐transferrin receptor antibody for 2 h, and anti‐rabbit IgG (Alexa Fluor 488‐conjugated) for 1 h. The fluorescent images were captured by confocal laser scanning microscopy (CLSM, LSM 880).

### The Quantitative Analysis of Iron Internalized in Three Cell Lines

1×10^5^ 3T3, MDA‐MB‐231, and MCF‐7 cells were seeded on 12‐well plates and incubated with T7‐MNTs and MNP@SiO_2_ (50 µg mL^−1^ of Fe content) for 24 h, respectively. Afterwards, the numbers of all cells in different treatment groups were collected and counted. The quantification of intracellular Fe contents in different groups were detected by inductively coupled plasma optical emission spectroscopy (ICP‐OES).

### Bio‐TEM Analysis

2×10^5^ MDA‐MB‐231 and MCF‐7 cells were seeded on each dish (60 mm) for 24 h, respectively. In the groups of “MF‐” and “MF+”, cells were incubated with T7‐MNTs (50 µg mL^−1^ of Fe content) for 24 h, respectively. For the RSL3 group, cells were treated with RSL3 (20 µm) for 24 h. Next, the “MF+” group was treated with MF (260 mT,15 Hz) for 30 min. Afterwards, cells were collected and fixed by 2.5% glutaraldehyde. After dehydrating, cells were embedded in Epon Araldite resin. The ultrathin sections of 50 nm were performed for TEM image observation.

### Colocalization Experiment

6×10^4^ MDA‐MB‐231 and MCF‐7 cells were seeded on each confocal dish (25 mm) before being incubated with FITC labeled T7‐MNTs (FITC@T7‐MNTs, 50 µg mL^−1^ of Fe content) for 24 h, aside from the control group. Then, cells were stained by Lyso‐Tracker Red (1 µm), Mito‐Tracker Red (1 µm) and ER‐Tracker Red (1 µm), respectively. Finally, cells were stained by Hoechst 33 342 for 30 min. For the F‐actin staining experiment, cells were fixed for 10 min in 4% paraformaldehyde and treated with PBS containing 0.1% Triton X‐100 for 10 min, and then stained with Actin‐Tracker Red for 30 min and DAPI for 10 min, respectively. The confocal fluorescent images were captured by CLSM.

### Lysosomal Membrane Permeabilization Assessment

6×10^4^ MDA‐MB‐231 and MCF‐7 cells were seeded on each confocal dish for 24 h, respectively, and then transfected with EGFP‐Gal3 plasmid using lipofectamine 2000 reagent for another 24 h. Afterwards, T7‐MNTs (50 µg mL^−1^ of Fe content) were co‐incubated for 24 h. The “MF+” group was treated with MF (260 mT, 15 Hz) for 30 min. The cells incubated with free T7‐MNTs were used as the “MF‐” group, and the untreated cells were used as the control group. After MF treatment, the fluorescence images were observed under a live cell imaging microscope (EVOS, Life Technologies).

### The Intercellular Iron Distribution Staining

6×10^4^ MDA‐MB‐231 and MCF‐7 cells were cultured on each confocal dish for 24 h and co‐cultured with T7‐MNTs (50 µg mL^−1^ of Fe content) for 24 h. After MF stimulation, all cells were stained by Lyso‐Tracker Red (1 µm) and FerroOrange (1 µm) for 30 min orderly at 37°C. The fluorescence images were observed by CLSM.

### Cellular Labile Iron Measurement

1×10^5^ MDA‐MB‐231 and MCF‐7 cells were seeded on each dish (35 mm) for 24 h. After incubating with T7‐MNTs (50 µg mL^−1^ of Fe content) for 24 h, DFO (50 µm) was added in the “MF+DFO” group and incubated for 6 h before MF stimulation. After washing with PBS, cells were stained with Calcein‐AM (1 µm) for 30 min. Afterwards, cells were digested by trypsin and collected in the centrifuge tube. Finally, the cell suspension of each group was analyzed by using a CytoFLEX LX cytometer with a 488 nm argon laser.

### Cellular Iron Content Measurement

2×10^5^ MDA‐MB‐231 and MCF‐7 cells were seeded on each dish (60 mm) for 24 h and then incubated with T7‐MNTs (50 µg mL^−1^ of Fe content) for 24 h. Then, DFO (50 µM) was added in the “MF+DFO” group for 6 h before MF stimulation (260 mT, 15 Hz, 30 min). Cell lysis supernatants were collected and measured by the Iron Assay Kit (ab83366, Abcam).

### Lysosomal Acidity Detection

1×10^5^ MDA‐MB‐231 and MCF‐7 cells were seeded on each dish (35 mm) for 24 h and co‐incubated with T7‐MNTs (50 µg mL^−1^ of Fe content) for an additional 24 h. After MF application (260 mT, 15 Hz, 30 min), cells were stained by LysoSensor Yellow/Blue DND‐160 (PDMPO) (1 µM) for 15 min. Finally, the mean fluorescence intensities were measured by CytoFLEX LX.

### ROS Level Measurement

6×10^4^ MDA‐MB‐231 and MCF‐7 cells were seeded on each confocal dish for 24 h. Cells were incubated with T7‐MNTs (50 µg mL^−1^ of Fe content) or RSL3 (20 µm) for 24 h, respectively. Lip‐1 (1 µm), Fer‐1 (5 µm), and DFO (50 µm) were pre‐cultured for 6 h before the MF stimulation (260 mT, 15 Hz, 30 min). Then, cells were stained by DCFH‐DA probes (10 µm, 30 min). At last, all the samples were observed by a live cell imaging microscope.

### Liperfluo Staining

1×10^5^ MDA‐MB‐231 and MCF‐7 cells were seeded on each dish (35 mm) for 24 h, and then incubated with T7‐MNTs (50 µg/mL of Fe content) or RSL3 (20 µm) for 24 h. After incubating with Lip‐1 (1 µm), Fer‐1 (5 µm), and DFO (50 µm) for 6 h, the “MF+”, “MF+Lip‐1”, “MF+Fer‐1”, and “MF+DFO” groups were treated with MF stimulation (260 mT, 15 Hz, 30 min). All the groups were stained by Liperfluo (10 µm) for 30 min. At last, cells were collected and analyzed by CytoFLEX LX.

### MDA Level Measurement

1×10^5^ MDA‐MB‐231 and MCF‐7 cells were seeded on each dish (35 mm) for 24 h, and followed by the incubation of T7‐MNTs (50 µg mL^−1^ of Fe content) or RSL3 (20 µM) for 24 h. Afterwards, Lip‐1 (1 µm), Fer‐1 (5 µm), and DFO (50 µm) were pre‐cultured for 6 h before the MF application (260 mT, 15 Hz, 30 min). Finally, cells were collected and extracted the protein. MDA level was measured by the MDA kit.

### GSH Level and GPX4 Activity Measurement

1×10^5^ MDA‐MB‐231 and MCF‐7 cells seeded on each dish (35 mm) for 24 h. Then, “MF‐” group was incubated with T7‐MNTs (50 µg mL^−1^ of Fe content) for 24 h. The “MF+” group was incubated with T7‐MNTs (50 µg mL^−1^ of Fe content) for 24 h and followed by MF application (260 mT, 15 Hz, 30 min), cells without any treatment were used as the control group. Afterwards, the numbers of all cells in different treatment groups were collected and counted. The total GSH content and GPX4 activity were measured by the total glutathione assay kit and total glutathione peroxidase assay kit, respectively.

### Western Blotting Assay

1×10^5^ MDA‐MB‐231 and MCF‐7 cells were seeded on culture dishes (35 mm) for 24 h. Then, cells were incubated with T7‐MNTs (50 µg mL^−1^ of Fe content) or RSL3 (20 µm) for 24 h, respectively. The “MF+” and “MF+DFO” groups were treated with MF (260 mT, 15 Hz) for 30 min. DFO (50 µm) was pre‐cultured for 6 h before the MF stimulation. The untreated cells were used as the control group. Then, cells were collected and extracted the protein. Afterwards, the protein was subjected to sodium dodecyl sulphate‐polyacrylamide gel electrophoresis (SDS‐PAGE) and transferred onto the nitrocellulose membrane. After blocking with the 5% milk, the anti‐glutathione peroxidase 4 (1:1000 dilution), the anti‐cyclooxygenase 2 (1:1000 dilution), the anti‐xCT (1:1000 dilution), and the anti‐ferritin (1:1000 dilution) as the primary antibody were incubated with the nitrocellulose membrane at 4°C, respectively. After purification, the secondary antibody was co‐cultured with the nitrocellulose membrane. At last, the protein bands were observed by ChemiDoc XRS.

### Assessment of Ferroptosis

6×10^3^ MDA‐MB‐231 and MCF‐7 cells were seeded on the 96‐well plates, respectively. Then, cells were incubated with T7‐MNTs (50 µg mL^−1^ of Fe content) or RSL3 (20 µm) for 24 h. The “MF+Lip‐1”, “MF+Fer‐1”, and “MF+DFO” groups were respectively pre‐incubated with Lip‐1 (1 µm), Fer‐1 (5 µm), and DFO (50 µm) for 6 h, following by the MF stimulation (260 mT, 15 Hz, 30 min). Cell viability of all groups was measured by the CCK‐8 kit.

### Lipid Peroxide Measurement by C11‐BODIPY 581/591 Staining

6×10^4^ MDA‐MB‐231 and MCF‐7 cells were seeded on each confocal dish for 24 h. After incubating with T7‐MNTs (50 µg mL^−1^ Fe) or RSL3 (20 µm) for 24 h, the “MF+Lip‐1”, “MF+Fer‐1”, and “MF+DFO” groups were incubated in the presence of Lip‐1 (1 µm), Fer‐1 (5 µm), and DFO (50 µm) for 6 h. Then, the “MF+”, “MF+Lip‐1”, “MF+Fer‐1”, and “MF+DFO” groups were treated with MF stimulation (260 mT, 15 Hz, 30 min). All the groups were stained by BODIPY 581/591 C11 and Hoechst 33 342 orderly. The confocal images were captured by CLSM.

### Antitumor Therapy in Vivo

All animal experiments were approved by the animal protection and care committee of Tongji University. 2×10^6^ MDA‐MB‐231 cells were inoculated on the right groin of BALB/c nude mice (4 weeks old). The tumor volume was calculated as *V* (mm^3^) = 0.5×width^2^×length. The treatments were performed after the tumor volume reached ≈50 mm^3^. For 7 days experiment, the MDA‐MB‐231 subcutaneous tumor‐bearing mice were randomly separated into six groups (*n* = 6) : PBS, RSL3 (i.v., 3 mg kg^−1^), MF‐ (i.t., 5 mg kg^−1^ T7‐MNTs but without MF), MF+ (i.t., 5 mg kg^−1^ T7‐MNTs and treated with MF), MF+RSL3 (i.v., 3 mg kg^−1^ RSL3 and i.t., 5 mg kg^−1^ T7‐MNTs and treated with MF) and MF+Fer‐1+DFO (i.t., 5 mg kg^−1^ of T7‐MNTs and Fer‐1, respectively, and i.p., 20 mg kg^−1^ DFO, both treated with MF). The MF (260 mT, 15 Hz) was applied for 7 days with 30 min per day. The tumor volumes and body weights of each mouse were recorded daily. After 7 days treatment, the tumors of mice were performed for MDA level measurement and histological analysis. The organs of mice were collected for H&E staining. For 14 days experiment, the experimental groups were designed as eight groups (*n* = 6): PBS, RSL3, MF‐, MF+, MF+RSL3, MF+Fer‐1+DFO, MF+Fer‐1, MF+DFO. The MF+Fer‐1 group and MF+DFO group were injected with Fer‐1 (i.t., 5 mg kg^−1^) and DFO (i.p., 20 mg kg^−1^) every other day, respectively, following the T7‐MNTs treatment. The other groups were treated identically as 7 days experiment. During the treatment and observation period, tumor volume and body weight were measured every 2 days. All the mice were sacrificed and the tumor were collected for photographing on day 14.

### RT‐qPCR Analysis

Cells were placed in 1 mL TRIzol for RNA extraction. The tumor tissues were mechanically disrupted by surgical scissors in PBS, and then placed in 1 mL TRIzol for RNA extraction. Reverse transcription was conducted by the Reverse Transcription System cDNA synthesis kit (Takara, Japan). RT‐qPCR was conducted by the FastStart Universal SYBR Green Master Mix (ROX) (Takara, Japan). The results were normalized to β‐actin and analyzed through the relative Ct method (2‐ΔΔCt). The primer sequences of genes are listed as follows: GPX4, GGAGCCAGGGAGTAACGAAG (forward), ACGGTGTCCAAACTTGGTGAA (reverse); CHAC1, GCTGTGGATTTTCGGGTACG (forward), CACACGGCCAGGCATCTT (reverse); SLC7A11, TCCTGCTTTGGCTCCATGAACG (forward), AGAGGAGTGTGCTTGCGGACAT (reverse); ACSL4, GCTATCTCCTCAGACACACCGA (forward), AGGTGCTCCAACTCTGCCAGTA (reverse); PTGS2, CGGTGAAACTCTGGCTAGACAG (forward), GCAAACCGTAGATGCTCAGGGA (reverse); TFRC, GGCTACTTGGGCTATTGTAAAGG (forward), CAGTTTCTCCGACAACTTTCTCT (reverse); FTH1, AGAACTACCACCAGGACTCAGA (forward), GTCAAAGTAGTAAGACATGGACAG (reverse).

### Statistical Analysis

One‐way ANOVA analysis, two‐way ANOVA analysis, and T‐test were performed for the statistical analysis by GraphPad Prism 9. The numerical data were given as Mean ± SD. The p values were set at **** *p* < 0.0001, *** *p* < 0.001, ** *p* < 0.01, and * *p* < 0.05.

## Conflict of Interest

The authors declare no conflict of interest.

## Supporting information

Supporting Information

Supplemental Movie 1

## Data Availability

The data that support the findings of this study are available from the corresponding author upon reasonable request.

## References

[1] a) S. J. Dixon , K. M. Lemberg , M. R. Lamprecht , R. Skouta , E. M. Zaitsev , C. E. Gleason , D. N. Patel , A. J. Bauer , A. M. Cantley , W. S. Yang , B. Morrison , B. R. Stockwell , Cell 2012, 149, 1060; 22632970 10.1016/j.cell.2012.03.042 PMC3367386

[2] X. Chen , R. Kang , G. Kroemer , D. Tang , Nat. Rev. Clin. Oncol. 2021, 18, 280. 33514910 10.1038/s41571-020-00462-0

[3] S. J. Dixon , B. R. Stockwell , Nat. Chem. Biol. 2014, 10, 9. 24346035 10.1038/nchembio.1416

[4] a) F. Rizzollo , S. More , P. Vangheluwe , P. Agostinis , Trends. Biochem. Sci. 2021, 46, 960; 34384657 10.1016/j.tibs.2021.07.003

[5] a) T. T. Mai , A. Hamaï , A. Hienzsch , T. Cañeque , S. Müller , J. Wicinski , O. Cabaud , C. Leroy , A. David , V. Acevedo , A. Ryo , C. Ginestier , D. Birnbaum , E. Charafe‐Jauffret , P. Codogno , M. Mehrpour , R. Rodriguez , Nat. Chem. 2017, 9, 1025; 28937680 10.1038/nchem.2778 PMC5890907

[6] a) K. Li , C. Lin , M. Li , K. Xu , Y. He , Y. Mao , L. Lu , W. Geng , X. Li , Z. Luo , K. Cai , ACS Nano 2022, 16, 2381; 35041395 10.1021/acsnano.1c08664

[7] a) H. Yuan , Z. Han , Y. Chen , F. Qi , H. Fang , Z. Guo , S. Zhang , W. He , Angew.Chem. Int. Ed. 2021, 60, 8174; 10.1002/anie.202014959 33656228

[8] W. Li , S. Yin , Y. Shen , H. Li , L. Yuan , X.‐B. Zhang , J. Am. Chem. Soc. 2023, 145, 3736. 36730431 10.1021/jacs.2c13222

[9] a) L. Wang , X. Zhang , Z. You , Z. Yang , M. Guo , J. Guo , H. Liu , X. Zhang , Z. Wang , A. Wang , Y. Lv , J. Zhang , X. Yu , J. Liu , C. Chen , Angew.Chem. Int. Ed. 2022, 135, e202217448; 10.1002/anie.202217448 36585377

[10] a) J. An , H. Hong , M. Won , H. Rha , Q. Ding , N. Kang , H. Kang , J. S. Kim , Chem. Soc. Rev. 2023, 52, 30; 36511945 10.1039/d2cs00546h

[11] a) M. H. Cho , E. J. Lee , M. Son , J.‐H. Lee , D. Yoo , J.‐W. Kim , S. W. Park , J.‐S. Shin , J. Cheon , Nat. Mater. 2012, 11, 1038; 23042417 10.1038/nmat3430

[12] J.‐u. Lee , W. Shin , Y. Lim , J. Kim , W. R. Kim , H. Kim , J.‐H. Lee , J. Cheon , Nat. Mater. 2021, 20, 1029. 33510447 10.1038/s41563-020-00896-y

[13] a) Q. Yu , B. Zhang , Y.‐M. Zhang , Y.‐H. Liu , Y. Liu , ACS Appl. Mater. Interfaces 2020, 12, 13709; 32118400 10.1021/acsami.0c01762

[14] a) Y. Chen , P. Han , Y. Wu , Z. Zhang , Y. Yue , W. Li , M. Chu , Small 2018, 14, 1802799; 10.1002/smll.201802799 30294915

[15] B. Yu , B. Choi , W. Li , D.‐H. Kim , Nat. Commun. 2020, 11, 3637. 32686685 10.1038/s41467-020-17380-5 PMC7371635

[16] a) Y. Shen , W. Zhang , G. Li , P. Ning , Z. Li , H. Chen , X. Wei , X. Pan , Y. Qin , B. He , Z. Yu , Y. Cheng , ACS Nano 2021, 15, 20020; 34807565 10.1021/acsnano.1c07615

[17] J. Wu , P. Ning , R. Gao , Q. Feng , Y. Shen , Y. Zhang , Y. Li , C. Xu , Y. Qin , G. R. Plaza , Q. Bai , X. Fan , Z. Li , Y. Han , M. S. Lesniak , H. Fan , Y. Cheng , Adv. Sci. 2020, 7, 1902933. 10.1002/advs.201902933 PMC7312334 32596106

[18] J. H. Lee , J. A. Engler , J. F. Collawn , B. A. Moore , Eur. J. Biochem. 2001, 268, 2004. 11277922 10.1046/j.1432-1327.2001.02073.x

[19] Y. I. Golovin , S. L. Gribanovsky , D. Y. Golovin , N. L. Klyachko , A. G. Majouga , ?L. M. Master , M. Sokolsky , A. V. Kabanov , J. Controlled Release 2015, 219, 43. 10.1016/j.jconrel.2015.09.038 PMC4841691 26407671

[20] S. V. Torti , F. M. Torti , Nat. Rev. Cancer 2013, 13, 342. 23594855 10.1038/nrc3495 PMC4036554

[21] a) J. J. Rennick , A. P. R. Johnston , R. G. Parton , Nat. Nanotechnol. 2021, 16, 266; 33712737 10.1038/s41565-021-00858-8

[22] D. L. Fogde , C. P. R. Xavier , K. Balnyte , L. K. K. Holland , K. Stahl‐Meyer , C. Dinant , E. Corcelle‐Termeau , C. Pereira‐Wilson , K. Maeda , M. Jaattelä , Cells 2022, 11, 4079. 36552844 10.3390/cells11244079 PMC9776894

[23] G. Miotto , M. Rossetto , M. L. Di Paolo , L. Orian , R. Venerando , A. Roveri , A.‐M. Vuckovic , V. Bosello Travain , M. Zaccarin , L. Zennaro , M. Maiorino , S. Toppo , F. Ursini , G. Cozza , Redox Biol. 2020, 28, 101328. 31574461 10.1016/j.redox.2019.101328 PMC6812032

[24] a) C. Xue , M. Li , C. Liu , Y. Li , Y. Fei , Y. Hu , K. Cai , Y. Zhao , Z. Luo , Angew. Chem., Int. Ed. 2021, 60, 8938; 10.1002/anie.202016872 33543529

[25] a) L. Zhou , C. Dong , L. Ding , W. Feng , L. Yu , X. Cui , Y. Chen , Nano Today 2021, 39, 101212;

[26] a) S. Yang , Y. Wu , W. Zhong , R. Chen , M. Wang , M. Chen , Adv. Mater. 2023, 2304098; 10.1002/adma.202304098 37689975

[27] Z. Zhou , J. Song , L. Nie , X. Chen , Chem. Soc. Rev. 2016, 45, 6597. 27722328 10.1039/c6cs00271d PMC5118097

[28] a) S. Liang , J. Yao , D. Liu , L. Rao , X. Chen , Z. Wang , Adv. Mater. 2023, 35, 2211130; 10.1002/adma.202211130 36881527

[29] A. Serrano‐Puebla , P. Boya , Biochem. Soc. Trans. 2018, 46, 207. 29472365 10.1042/BST20170130

[30] a) K. Bersuker , J. M. Hendricks , Z. Li , L. Magtanong , B. Ford , P. H. Tang , M. A. Roberts , B. Tong , T. J. Maimone , R. Zoncu , M. C. Bassik , D. K. Nomura , S. J. Dixon , J. A. Olzmann , Nature 2019, 575, 688; 31634900 10.1038/s41586-019-1705-2 PMC6883167

[31] C. Xu , S. Sun , T. Johnson , R. Qi , S. Zhang , J. Zhang , K. Yang , Cell Rep. 2021, 35, 109235. 34133924 10.1016/j.celrep.2021.109235

[32] F. Ursini , M. Maiorino , Free. Radic. Biol. Med. 2020, 152, 175. 32165281 10.1016/j.freeradbiomed.2020.02.027

[33] B. R. Stockwell , Cell 2022, 185, 2401. 35803244 10.1016/j.cell.2022.06.003 PMC9273022

[34] a) S. Doll , B. Proneth , Y. Y. Tyurina , E. Panzilius , S. Kobayashi , I. Ingold , M. Irmler , J. Beckers , M. Aichler , A. Walch , H. Prokisch , D. Trümbach , G. Mao , F. Qu , H. Bayir , J. Füllekrug , C. H. Scheel , W. Wurst , J. A. Schick , V. E. Kagan , J. P. F. Angeli , M. Conrad , Nat. Chem. Biol. 2017, 13, 91; 27842070 10.1038/nchembio.2239 PMC5610546

[35] M. Chen , J. Wu , P. Ning , J. Wang , Z. Ma , L. Huang , G. R. Plaza , Y. Shen , C. Xu , Y. Han , M. S. Lesniak , Z. Liu , Y. Cheng , Small 2020, 16, 1905424. 10.1002/smll.201905424 31867877

[36] a) N. Zhao , L. Yan , J. Xue , K. Zhang , F.‐J. Xu , Nano Today 2021, 38, 101118;

