# Supplementary material for: A Lysosome‐Targeted Magnetic Nanotorquer Mechanically Triggers Ferroptosis for Breast Cancer Treatment
Source: Adv Sci (Weinh). 2023 Dec 14;11(9):2302093. doi: 10.1002/advs.202302093 (PMC10916606; doi:10.1002/advs.202302093)
Supplement: Supplementary file 1 — Supporting Information [file ADVS-11-2302093-s002.pdf]

ADVANCED SCIENCE
Open Access

Supporting Information

for *Adv. Sci.*, DOI 10.1002/advs.202302093

A Lysosome-Targeted Magnetic Nanotorquer Mechanically Triggers Ferroptosis for Breast Cancer Treatment

*Xueyan Wei, Yingze Li, Haotian Chen, Rui Gao, Peng Ning, Yingying Wang, Wanxin Huang, Erzhen Chen, Lan Fang, Xingrong Guo, Cheng Lv and Yu Cheng**

Supporting Information

A Lysosome-Targeted Magnetic Nanotorquer Mechanically Triggers Ferroptosis for Breast Cancer Treatment

*Xueyan Wei, Yingze Li, Haotian Chen, Rui Gao, Peng Ning, Yingying Wang, Wanxin Huang, Erzhen Chen, Lan Fang, Xingrong Guo, Cheng Lv, Yu Cheng**

X. Wei, Y. Li, H. Chen, R. Gao, P. Ning, Y. Wang, W. Huang, E. Chen, C. Lv, Prof. Y. Cheng
Translational Research Institute of Brain and Brain-Like Intelligence, Shanghai Fourth People's Hospital, School of Medicine, Tongji University, Shanghai, 200092, China
E-mail: yucheng@tongji.edu.cn

L. Fang
Shanghai Tenth People's Hospital, School of Medicine, Tongji University Cancer Center, Shanghai, 200072, China

X. Guo
Hubei Key Laboratory of Embryonic Stem Cell Research, Hubei Clinical Research Center for Umbilical Cord Blood Hematopoietic Stem Cells, Taihe Hospital, Hubei University of Medicine, Shiyan, Hubei, 442000, China

Materials and Reagents. Ferric (III) acetylacetonate ($Fe(acac)_3$), deferoxamine (DFO), dibenzyl ether, and zinc acetylacetonate hydrate ($Zn(acac)_2$) were purchased from Sigma. Oleic acid (AR), N-hydroxy succinimide (NHS), cyclohexane (AR), ammonium hydroxide solution (AR), chlorpromazine and 1-(3-Dimethylaminopropyl)-3-ethyl carbodiimide methiodide (EDC) were purchased from Aladdin. Tetraethyl orthosilicate (TEOS, 98%) and Igepal CO-520 were purchased from Macklin. Silane-PEG 2000-NHS was purchased from Ponsure biological. T7 peptide (98%, MW 892.5) was purchased from GL Biochem (Shanghai) Ltd. Prussian blue iron stain kit was bought from Beijing Solarbio Science & Technology Co., Ltd. EGFP-Gal3 plasmid was sourced from Systems Biosciences. Lipofectamine 2000 reagent and BODIPY™ 581/591 C11 were purchased from Thermo Scientific (China). Liproxstatin-1 (Lip-1), RSL3, and ferrostatin-1 (Fer-1) were purchased from Selleck. Anti-glutathione peroxidase 4 (ab125066), anti-transferrin receptor (ab84036), anti-cyclooxygenase 2 (ab179800), anti-xCT (ab307601), anti-ferritin (ab75973), and iron assay kit (ab83366) were purchased from Abcam (UK). Anti-rabbit IgG (Alexa Fluor® 488 Conjugate, 4412S) was purchased from Cell Signaling Technology. LysoSensor™ Yellow/Blue DND-160 (PDMPO) was bought from Yeasen (Shanghai). FerroOrange, liperfluo, and CCK-8 assay kit were bought from Dojindo laboratories (China). Lyso-Tracker Red, Lyso-Tracker Green, Mito-Tracker Red CMXRos, ER-Tracker Red, Actin-Tracker Red, DCFH-DA, Hoechst 33342, DAPI, Calcein-AM, malondialdehyde (MDA) assay kit, total glutathione assay kit, and total glutathione peroxidase assay kit were bought from Beyotime biotechnology. High glucose Dulbecco's Modified Eagle's Medium (DMEM) was purchased from Biosharp Life sciences (China). Penicillin streptomycin and fetal bovine serum were bought from Gibco (America).

Characterization of T7-MNTs. Transmission electron microscopy (TEM, JEOL JEM 2100F) and vibrating sample magnetometer (VSM, LakeShore7404, US) were performed for characterizing the morphological, structural, and magnetic features of T7-MNTs. T7 peptide modification was characterized by UV−vis spectrophotometer (Cary 60, Agilent). The hydrodynamic particle size and zeta potential of T7-MNTs and $MNP@SiO_2$ were detected by ZETA SIZER Nano series (Nano-ZS90, Malvern Ltd.).

MFG-100 equipment was used to record the magneto-mechanical properties of T7-MNTs. T7-MNTs were dispersed in cell culture medium with 25 μg/mL concentration of Fe and the MF with parameters of 40 mT, 0.5 Hz was applied.

Cell Culture and Cell Viability Assay. 3T3, MDA-MB-231, and MCF-7 cells were cultured in the medium (containing 90% high glucose DMEM medium, 10% FBS and 1% penicillin-streptomycin) and incubated at 5% CO_2, 37°C. 5×10^3 MDA-MB-231 and MCF-7 cells were seeded on 96-well plates, respectively. After 24 h, T7-MNTs at different concentrations (Fe = 0, 12.5, 25, 50, 100 μg/mL) were added to the plates. Cells without any treatment were used as the control group. Cell viability was measured after different incubation time (t = 24 h, 72 h, 120 h, 168 h).

Cellular Uptake of T7-MNTs and MNP@SiO₂ →

Cellular Uptake of T7-MNTs and MNP@SiO_2. 1×10^5 3T3, MDA-MB-231, and MCF-7 cells were seeded on 12-well plates for 24 h, respectively. Cells were incubated with T7-MNTs and MNP@SiO_2 (50 μg/mL of Fe content), respectively. After 24 h, cells were performed for Prussian blue iron staining and imaged under a live cell imaging system.

Endocytic Mechanisms of T7-MNTs. 6×10^4 MDA-MB-231 and MCF-7 cells were seeded on each confocal dish for 24 h. Then, the cells were pre-incubation of chlorpromazine (10 μg/mL) and excess anti-transferrin receptor antibodies (20 μg/mL) for 1 h at 37°C, respectively. Afterwards, FITC@T7-MNTs (50 μg/mL of Fe content) were co-incubated for 24 h. Cells treated with FITC@T7-MNTs only were used as controls. Finally, cells were stained by Lyso-Tracker Red (1 μM) and Hoechst 33342 orderly. The confocal fluorescent images were captured by CLSM.

LC-MS Analysis of Lipidomics. 1×10^7 MDA-MB-231 cells were seeded on 100 mm dish for 24 h. “MF+” and “MF-” groups were incubated with T7-MNTs (50 μg/mL of Fe content) for 24 h. Afterwards, the “MF+” group was treated with MF application (260 mT, 15 Hz, 30 min). Then, cells were digested by trypsin and collected in centrifuge tubes. After centrifuging at 1000 rpm for 3 min, the cell clusters were collected to extract the lipids. All the samples were examined by liquid chromatography-mass spectrometry (LC-MS) analysis. LC-MS analysis was performed by using an UHPLC-Q Exactive HF-X systerm (equipping with a Accucore

C30 column of 100 mm × 2.1 mm, 2.6 μm). 50% of acetonitrile-water sollution (containing 0.1% of formic acid and 10 mmol/L ammonium acetate) was used as the mobile phase A. The mobile phase B consisted of 10% acetonitrile, 88% isopropanol and 2% water. The washed gradient was as follows: 0 ~ 4 min, 65% ~ 40% A; 4 ~ 12 min, 40% ~ 15% A; 12 ~ 15 min, 15% ~ 0% A; 15 ~ 17 min, 0% A. The flow rate was 0.4 mL/min and the column temperature was maintain at 40 °C. The injection volume was 5 μL. All the samples were ionized by electrospray, and the mass spectrum signals were acquired by positive and negative modes (the spray voltage of 3.0 kV and -3.0 kV), respectively. The equipment conditions were set as following: scan type as 200-2000 (m/z), sheath gas flow rate as 60 psi, aux gas flow rate as 20 psi, aux gas heater temp as 370℃, normalized collision energy as 20-40-60 (v), respectively. The datas were analyzed by the Lipidsearch software (Thermo Fisher，USA).

The Cytotoxicity of T7-MNTs Treatment in Cancer and Normal Cell Lines. 6×10^3 A549, HepG2 and 3T3 cells were seeded on the 96-well plates, respectively. Then, the cells were incubated with T7-MNTs (50 μg/mL of Fe content) or RSL3 (20 μM) for 24 h. Afterwards, the “MF+” group was treated with MF stimulation (260 mT, 15 Hz, 30 min). Cells without any treatment were used as the control group. Cell viability of all groups was measured by the CCK-8 kit.

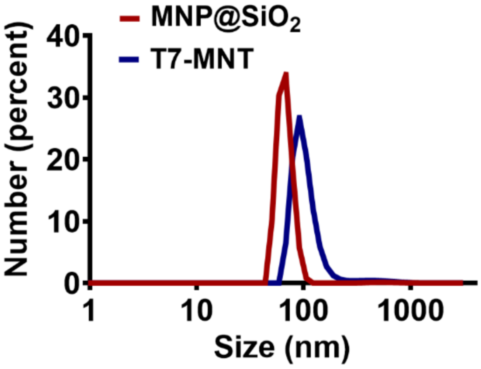

Figure S1. Dynamic light scattering (DLS) of MNP@SiO_2 and T7-MNTs.

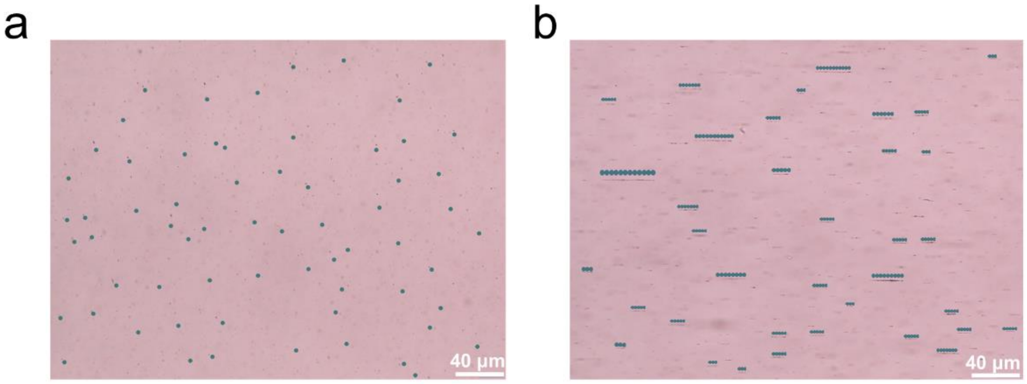

c

$$\tau = N \cdot \mathrm{mass}_{\mathrm{single}} \cdot (M_r + (M_0 - M_r) \cdot Cos\,\theta) \cdot B_0 \cdot Sin\theta$$

$$B_0 = 40\ mT, M_0 = 40.61\ emu/g,\ M_r = 0.26\ emu/g$$

$$For\ \theta \approx 45°,\ \tau_{\max} = N \cdot 1.82 \cdot pN \cdot \mu m,\ F_{\max} = \tau_{\max} / \frac{2L}{3}$$

$$For\ N = 5,\ F_{\max} = 36.4\ pN$$

Figure S2. Calculation of torques generated by T7-MNTs. T7-MNTs dispersed in a cell culture medium and assembled under the magnetic field. As B_0 = 40 mT, the single T7-MNT had a saturated magnetization of M_s = 40.61 emu/g and a remanent magnetization of M_r = 0.26 emu/g. The torques generated by T7-MNTs were calculated using the following equation:[1]

$$\tau = N \cdot mass_{single} \cdot (M_r + (M_0 - M_r) \cdot Cos\theta) \cdot B_0 \cdot Sin\theta$$

Where $mass_{single}$ is the mass of one magnetic nanotorquer. The θ is the angle between the direction of magnetic torque and the magnetic field. When the $\theta \approx 45°$,

a maximum value of the torques could be obtained and equaled to:

$$\tau_{\max} = N \cdot 1.82 \cdot pN \cdot \mu m$$

And the maximum torques may be estimated as $F_{\max} = \tau_{\max}$ /2L/3. For L = 375×10^{-3} μm. The forces induced by T7-MNTs were estimated to be 36.4 pN.

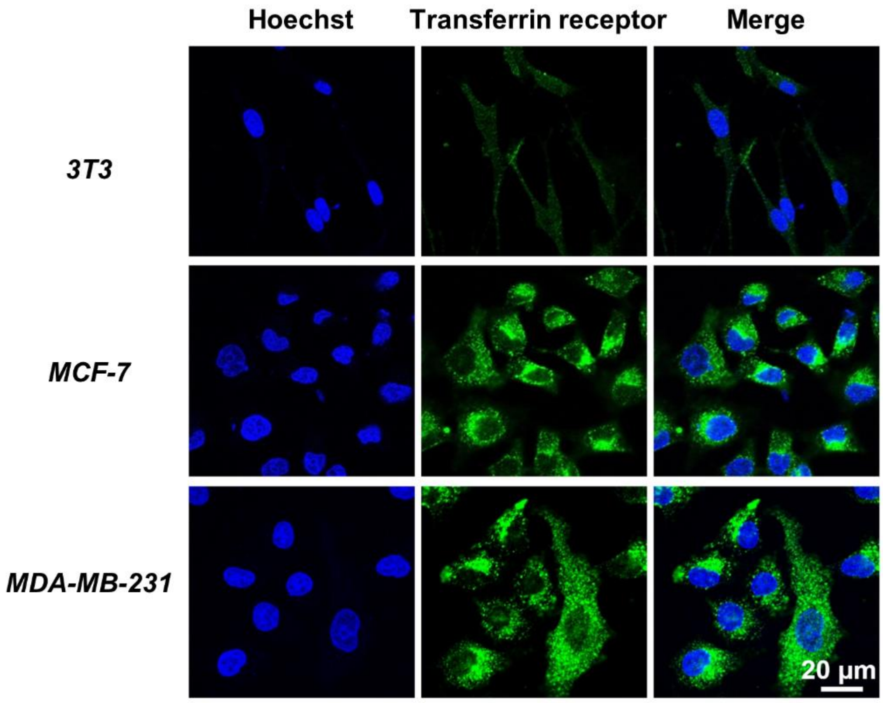

Figure S3. Transferrin receptors expression in normal and cancer cell lines. Confocal images of the expression of transferrin receptors on the normal cell (3T3 cells) and cancer cell (MCF-7 and MDA-MB-231 cells) membranes. Green fluorescence represents the staining of the transferrin receptors. Blue fluorescence represents nuclei stained by Hoechst. Scale bar: 20 μm.

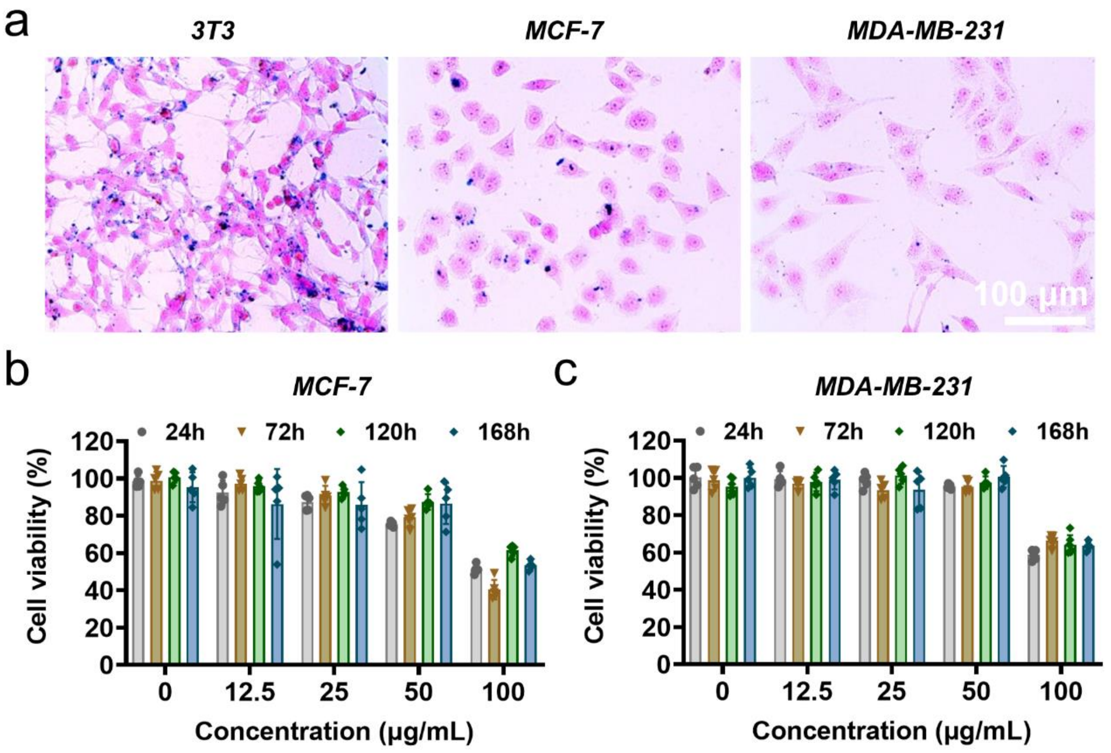

Figure S4. Uptake efficiency of MNP@SiO_2 in different cell lines and the cytotoxicity of T7-MNTs. (a) Prussian blue staining of MNP@SiO_2 co-incubated with 3T3, MCF-7, and MDA-MB-231 cells, respectively. Scale bar: 100 μm. (b-c) The cytotoxicity of T7-MNTs in MCF-7 (b) and MDA-MB-231 cells (c) treated with different concentrations (Fe = 0, 12.5, 25, 50, 100 μg/mL) and for different incubation time points (t = 24 h, 72 h, 120 h, 168 h). (n = 5). Cell viability was measured by CCK-8 kit.

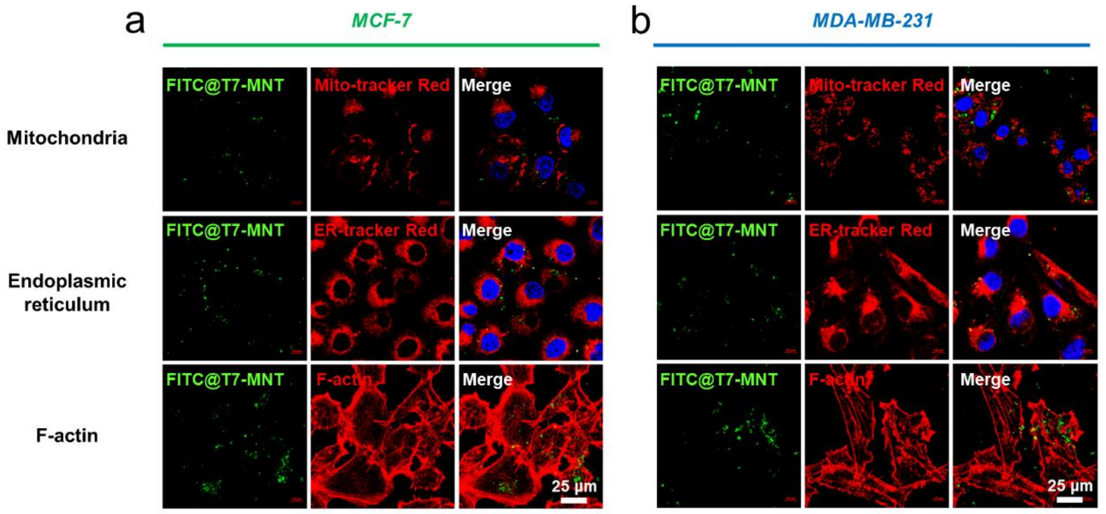

Figure S5. Intracellular distribution of T7-MNTs. Confocal fluorescence images of

FITC@T7-MNTs with mitochondria, endoplasmic reticulum, F-actin in MCF-7 (a) and MDA-MB-231 (b) cells, respectively. Cells were incubated with FITC@T7-MNTs (50 μg/mL of Fe content) for 24 h (scale bar: 25 μm).

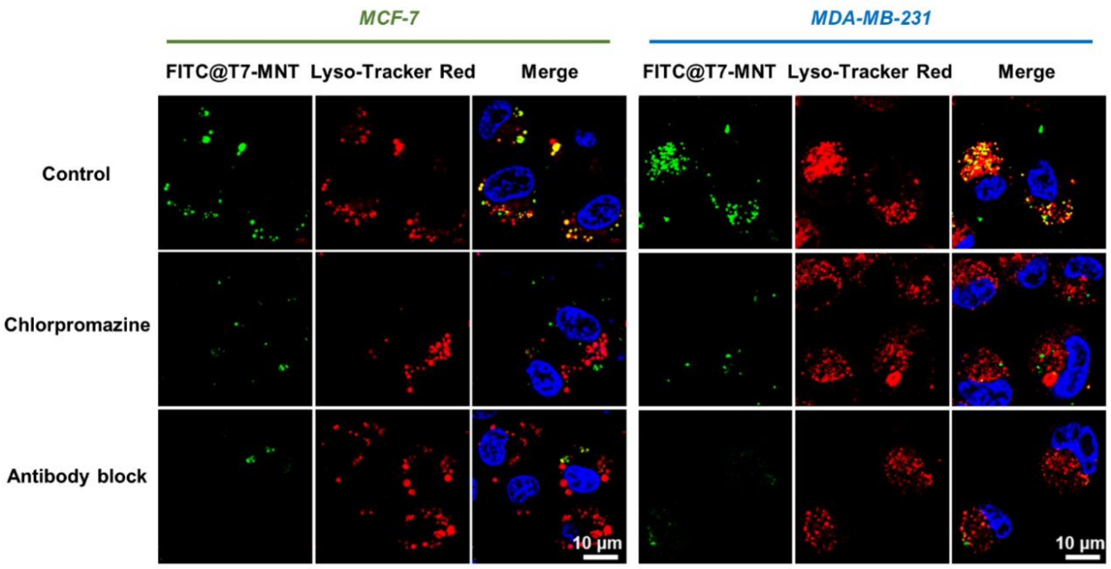

Figure S6. Effects of inhibitor and receptor blocking on the uptake of T7-MNTs. Confocal fluorescence images of FITC@T7-MNTs (50 μg/mL of Fe content) with pre-incubation of chlorpromazine (10 μg/mL) for 1 h at 37 °C. For receptor competitive experiment, excess anti-transferrin receptor antibodies (20 μg/mL) were added 1 h prior to the FITC@T7-MNTs. Cells treated with FITC@T7-MNTs (50 μg/mL of Fe content) only were used as controls. Scale bar: 10 μm.

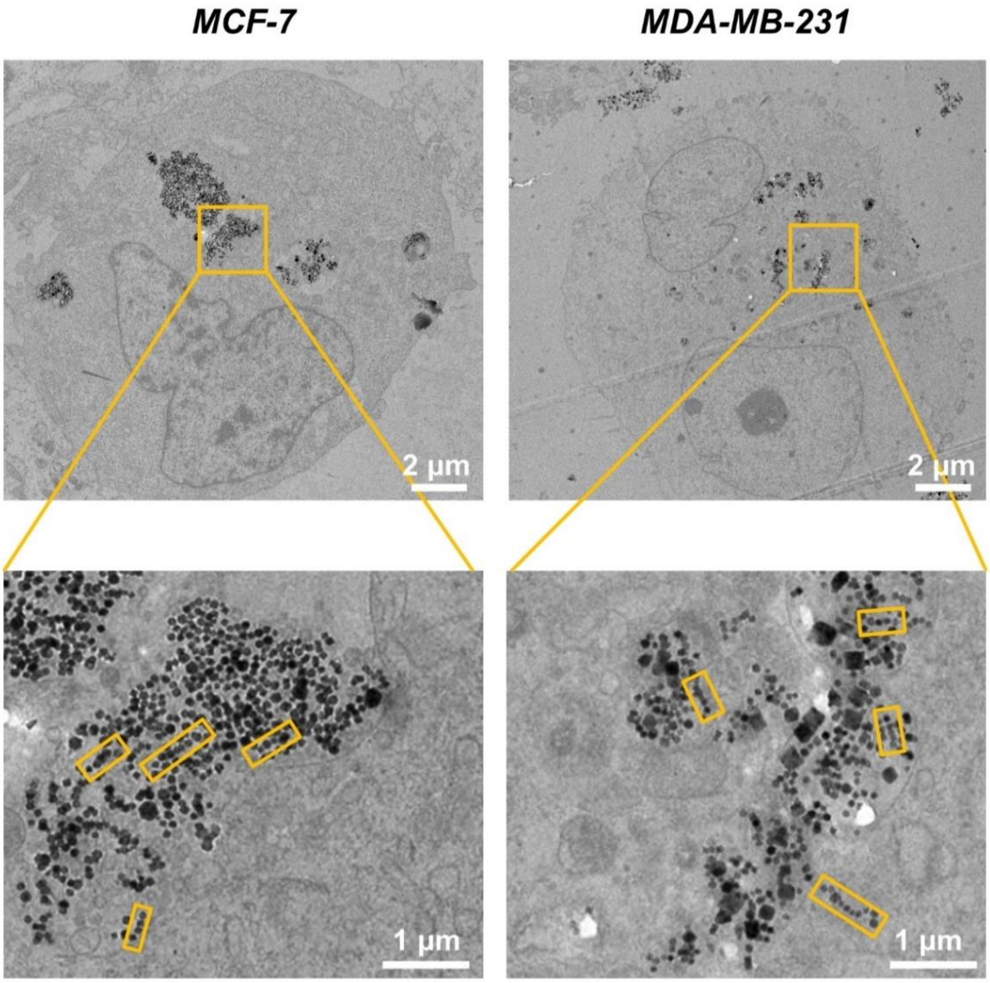

Figure S7. The assembly of T7-MNTs into rod-like swarms in lysosomes. The bio-TEM images of MCF-7 and MDA-MB-231 cells incubated with 50 µg/mL T7-MNTs for 24 h and followed by MF treatment (260 mT, 15 Hz, 30 min). The yellow box showed the T7-MNTs swarm structure. Scale bar: 2 µm and 1 µm.

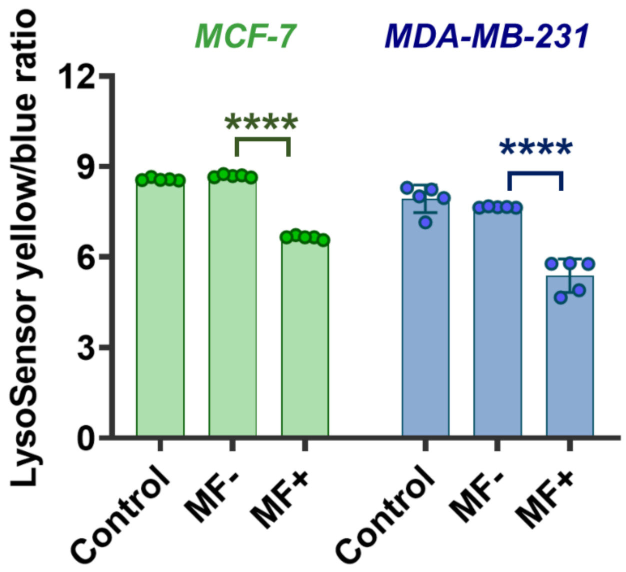

Figure S8. Evaluation of lysosomal integrity after T7-MNTs treatment. Flow cytometry analysis of the lysosomal acidity of MCF-7 and MDA-MB-231 cells by LysoSensor™ Yellow/Blue DND-160 probe (n = 5, ****$p < 0.0001$).

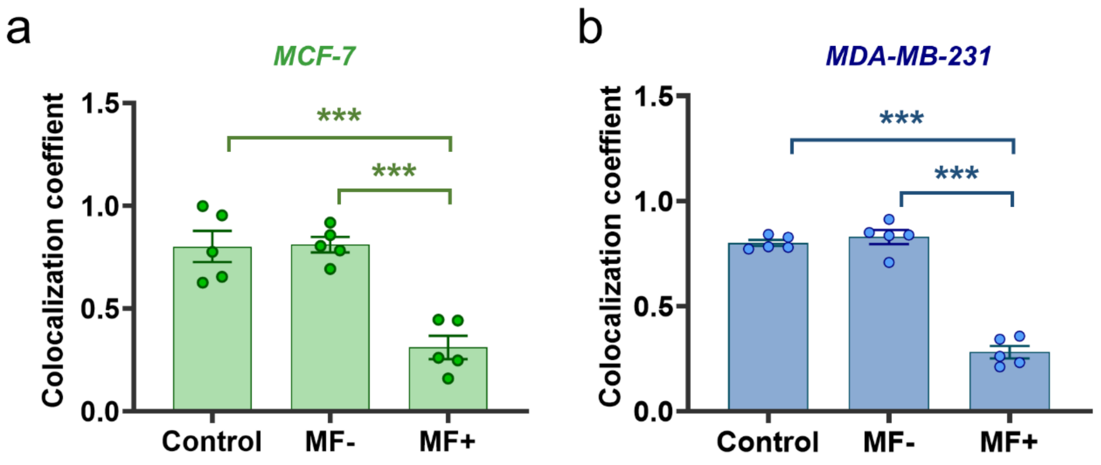

Figure S9. Colocalization coefficient of intracellular iron with lysosomes in MCF-7 and MDA-MB-231 cells. Histograms of tMr (Manders' overlap coefficient for red signals in thresholded image) corresponding to the signals of FerroOrange (red) and Lyso-Tracker Green, respectively (n = 5, ***$p < 0.001$, five randomly selected images were analyzed by Image J).

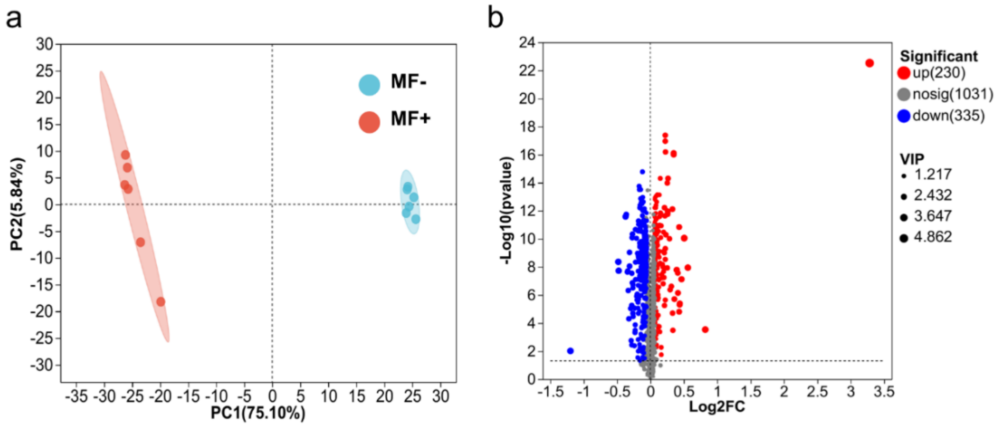

Figure S10. Lipid metabolomics analysis of T7-MNTs treatment. (a) PCA analysis of the different expression lipids profile in MDA-MB-231 cells with (MF+) and without T7-MNTs treatment (MF-). Axes in the PCA plot are relative to the two principal components with the largest variance. (b) Volcano plot of the different expression lipids in the MF- and MF+ groups.

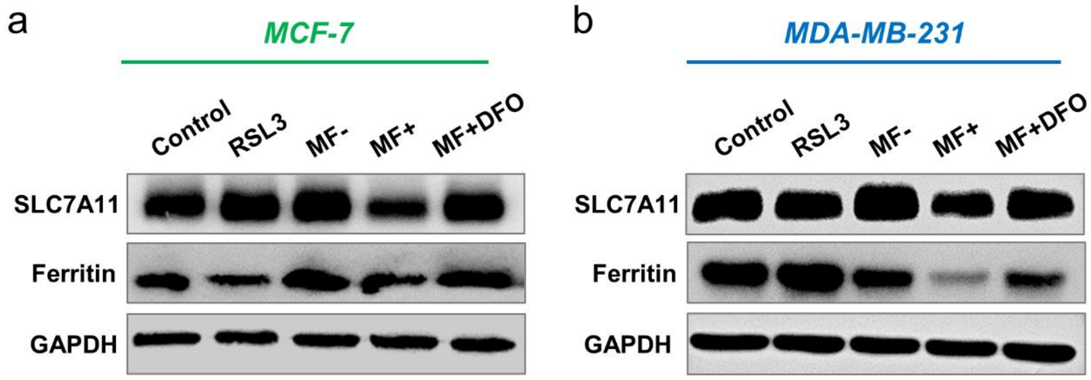

Figure S11. The expression level of SLC7A11 and ferritin with different treatment conditions in MCF-7 (a) and MDA-MB-231 cells (b). RSL3 group was treated with RSL3 (20 μM) for 24 h. The "MF+" group was treated with T7-MNTs and followed by MF application (260 mT, 15 Hz, 30 min). "MF+DFO" group were incubated with DFO (50 μM) for 6 h before MF application. The cells incubated with T7-MNTs were used as the "MF-" group, and untreated cells were used as the control group.

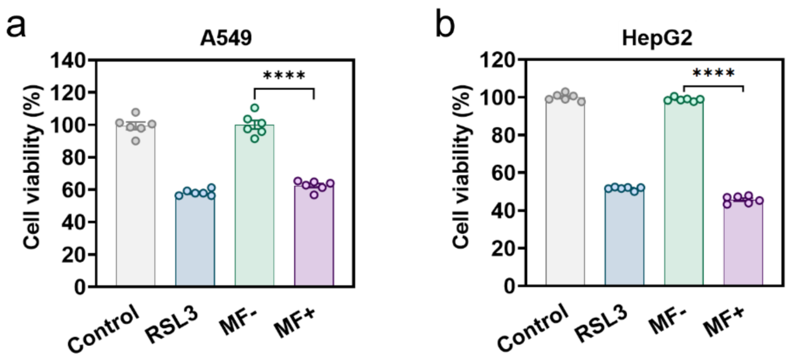

Figure S12. T7-MNTs-mediated cytotoxicity in A549 and HepG2 cells. Cell viability of A549 (a) and HepG2 cells (b) with different treatments (n = 6, ****p < 0.0001). The "MF+" group was treated with MF (260 mT, 15 Hz) for 30 min. 20 µM of RSL3 was used as a positive control. The cells incubated with T7-MNTs were used as the "MF-" group, and untreated cells were used as the control group. Cell viability was measured by CCK-8 kit.

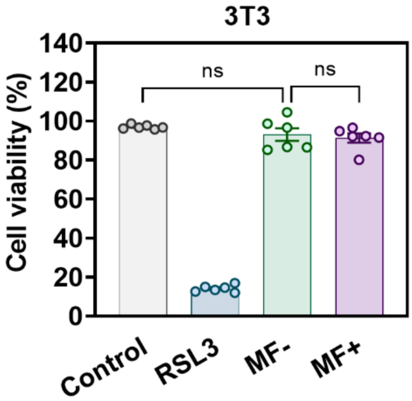

Figure S13. Cell viability of 3T3 cells under T7-MNTs treatment. The "MF+" group was treated with T7-MNTs and followed by MF application (260 mT, 15 Hz, 30 min). The cells incubated with free T7-MNTs were used as the "MF-" group, and untreated cells were used as the control group (n = 6, ****p < 0.0001, ns: no significance). Cell viability was measured by CCK-8 kit.

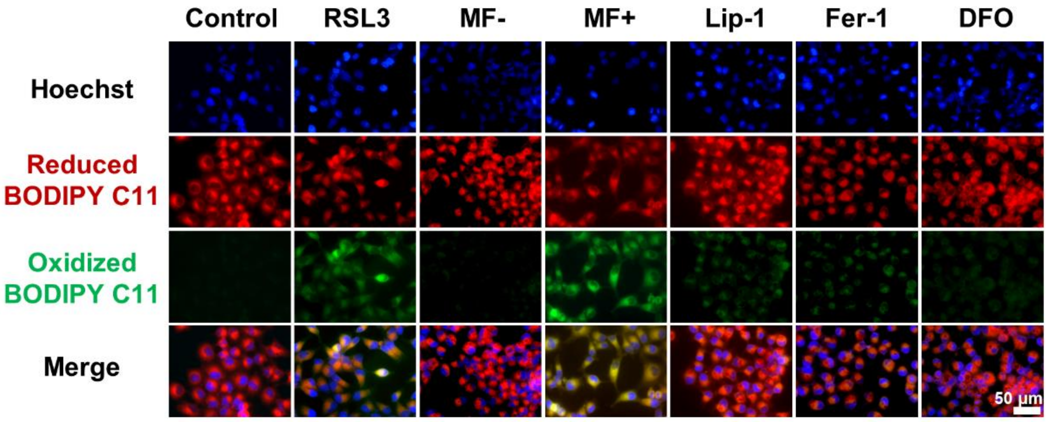

Figure S14. Ferroptosis induced by T7-MNTs in MDA-MB-231 cells. Lipid peroxidation of T7-MNTs incubated with MDA-MB-231 cells with different treatment conditions. Before MF stimulation (260 mT, 15 Hz, 30 min), the "MF+Lip-1", "MF+Fer-1", and "MF+DFO" groups were pre-treated with Lip-1 (1 μM), Fer-1 (5 μM), and DFO (50 μM) for 6 h, respectively. 20 μM of RSL3 was used as a positive control. Scale bar: 50 μm.

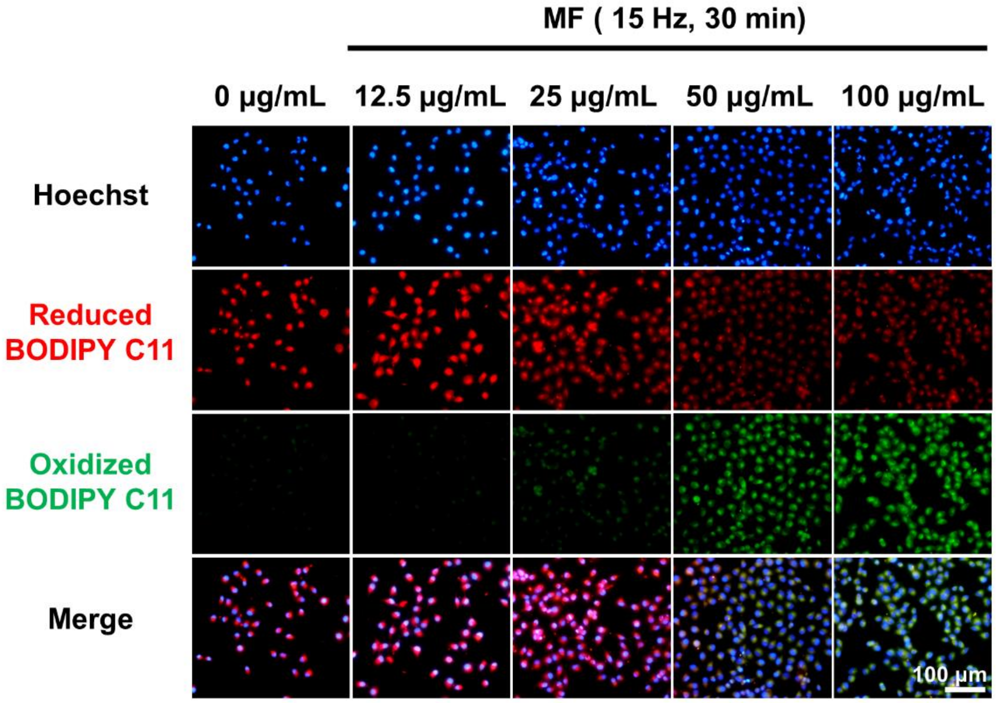

Figure S15. Lipid peroxidation in MDA-MB-231 cells treated with different concentrations of T7-MNTs (Fe = 0, 12.5, 25, 50, 100 μg/mL). MF was applied with the parameters of 260 mT, 15 Hz, and 30 min. Red fluorescence represents the reduced BODIPY C11 while green fluorescence represents the oxidized BODIPY C11. Blue

fluorescence represents nuclei stained by Hoechst. Scale bar: 100 μm.

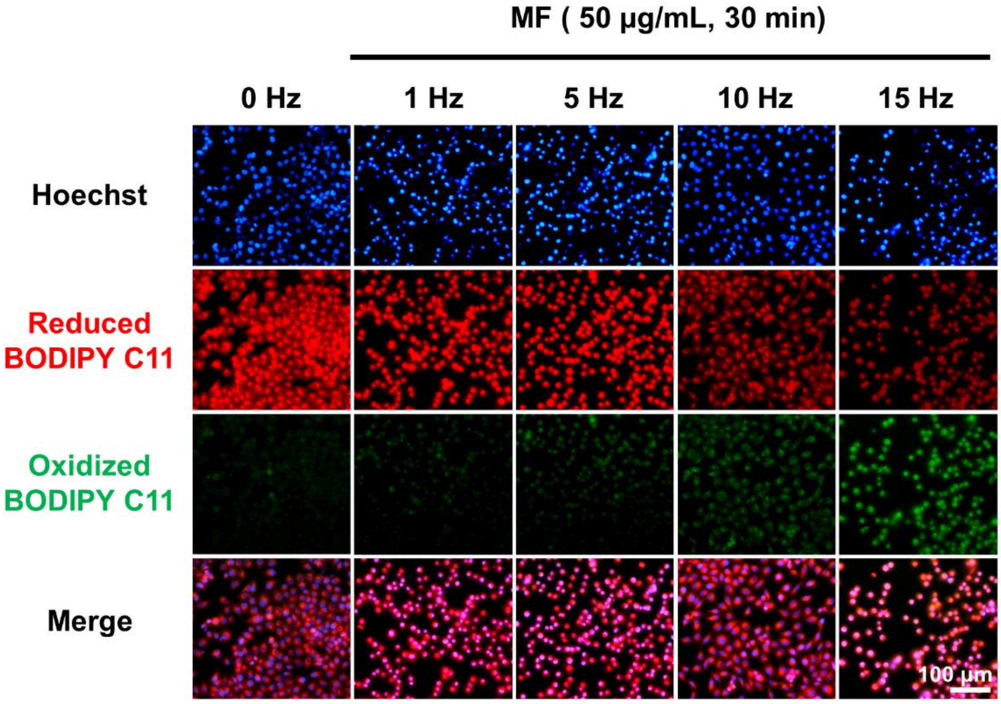

Figure S16. Lipid peroxidation in MDA-MB-231 cells with treatments under different frequencies of MF. The T7-MNTs concentration was 50 μg/mL, and the MF exposure time of 30 min. Scale bar: 100 μm.

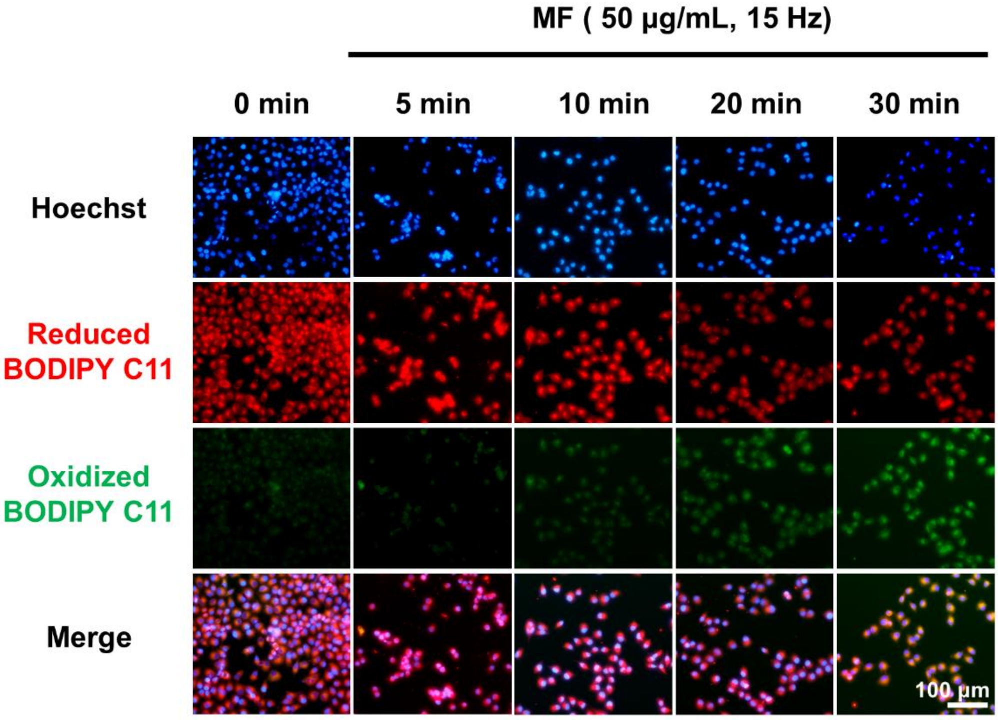

Figure S17. Lipid peroxidation in MDA-MB-231 cells with MF treatment at different periods. Cells treated with T7-MNTs at a Fe dose of 50 μg/mL. The frequency

of the MF application was 15 Hz. Scale bar: 100 µm.

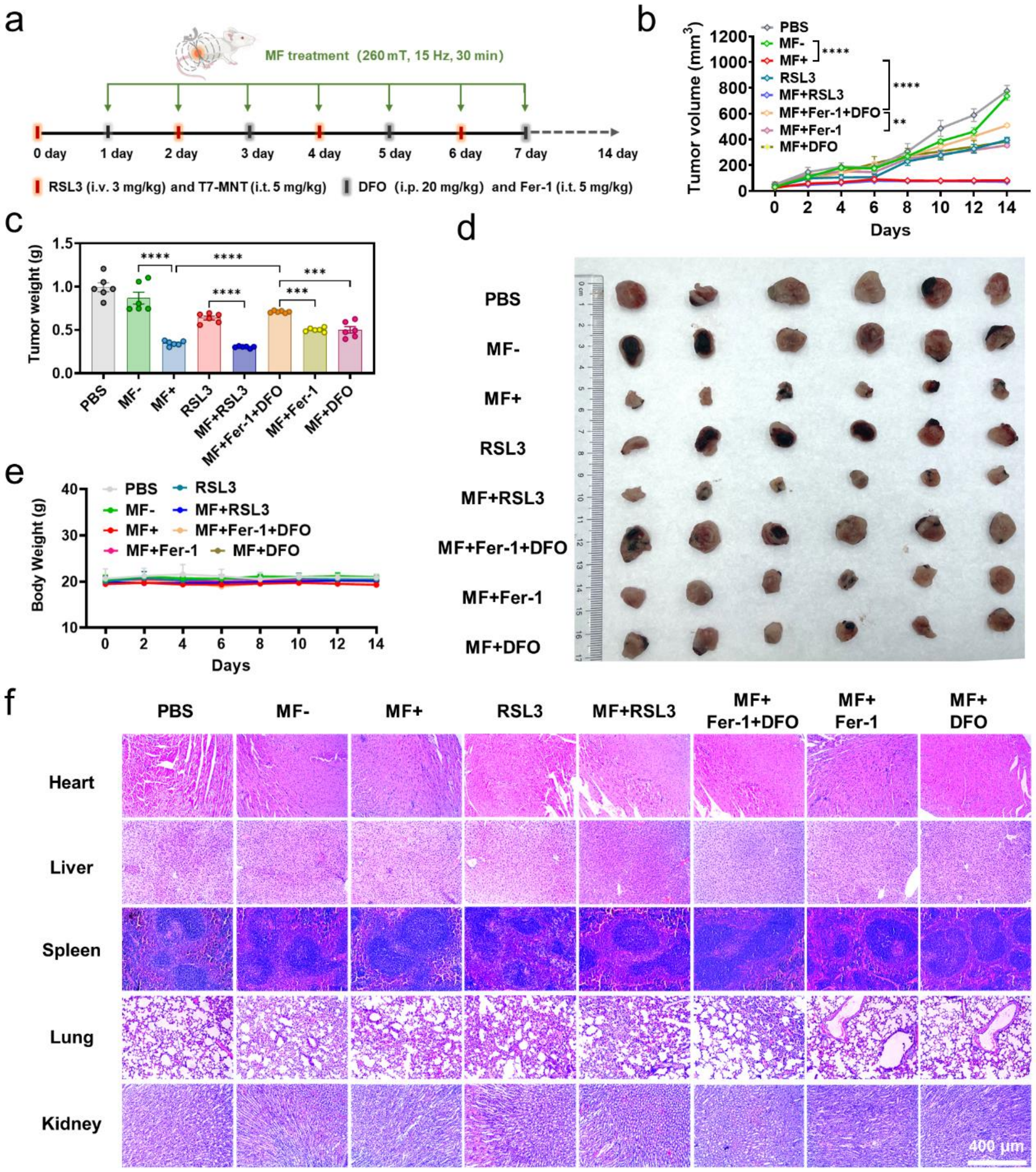

Figure S18. In vivo anticancer effect on MDA-MB-231 tumor-bearing mice. (a) Schematic diagram of the treatment process of MDA-MB-231-bearing mice. T7-MNTs (5 mg/kg, i.t.) and RSL3 (3 mg/kg, i.v.) were injected on days 0, 2, 4, and 6 for four times. Fer-1 (5 mg/kg, i.t.) and DFO (20 mg/kg, i.p.) were injected on days 1, 3, 5, and 7 for four times. The MF application (with therapeutic parameters of 260 mT, 15 Hz) was performed for 7 days with 30 min per day. (b) Tumor volume of mice recorded for 14 days from different treatment groups. (c) Tumor weight of each mouse was measured on day 14 (n = 6, ****$p < 0.0001$, ***$p < 0.001$). (d) Images of tumors on

day 14 in different groups. (e) Body weight of mice in different treatment groups. (f) H&E staining of major organs including heart, liver, spleen, lung, and kidney dissected from mice on day 14 after different treatments. Scale bar: 400 μm.

Video S1. The motion of T7-MNTs under MF exposure (40 mT, 0.5 Hz).

Reference

[1] M. W. Chen, J. J. Wu, P. Ning, J. J. Wang, Z. Ma, L. Q. Huang, G. R. Plaza, Y. J. Shen, C. Xu, Y. Han, M. S. Lesniak, Z. M. Liu, Y. Cheng, *Small* **2020**, 16, 1905424.
